# High selectivity of the hyperthermophilic subtilase propeptide domain toward inhibition of its cognate protease

**DOI:** 10.1128/spectrum.01487-23

**Published:** 2023-09-01

**Authors:** Miha Bahun, Nataša Poklar Ulrih

**Affiliations:** 1 Department of Food Science and Technology, Biotechnical Faculty, University of Ljubljana, Ljubljana, Slovenia; 2 Centre of Excellence for Integrated Approaches in Chemistry and Biology of Proteins (CIPKeBiP), Ljubljana, Slovenia; State Key Laboratory of Microbial Resources, Institute of Microbiology, Chinese Academy of Sciences, Beijing, China

**Keywords:** hyperthermophile, subtilase, propeptide, enzyme activation, enzyme inhibition, protein stability

## Abstract

Microbial extracellular subtilases are highly active proteolytic enzymes commonly used in commercial applications. These subtilases are synthesized in their inactive proform, which matures into the active protease under the control of the propeptide domain. In mesophilic bacterial prosubtilases, the propeptide functions as both an obligatory chaperone and an inhibitor of the subtilase catalytic domain. In contrast, the propeptides of hyperthermophilic archaeal prosubtilases act mainly as tight inhibitors and are not essential for subtilase folding. It is unclear whether this stronger inhibitory activity of hyperthermophilic propeptides results in their higher selectivity toward their cognate subtilases, in contrast to promiscuous mesophilic propeptides. Here, we showed that the propeptide of pernisine, a hyperthermostable archaeal subtilase, strongly interacts with and inhibits pernisine, but not the homologous subtilisin Carlsberg and proteinase K. Instead, the pernisine propeptide was readily degraded by subtilisin Carlsberg and proteinase K. In addition, the catalytic domain of unprocessed propernisine was also susceptible to degradation but became proteolytically stable after autoprocessing of propernisine into the inactive, noncovalent complex propeptide:pernisine. This allowed efficient transactivation of the autoprocessed complex propeptide:pernisine through degradation of pernisine propeptide by subtilisin Carlsberg and proteinase K at mesophilic temperature. Moreover, we demonstrated that active pernisine molecules are inhibited by the propeptide that is released after pernisine-catalyzed degradation of the unprocessed propernisine catalytic domain. This highlights the high inhibitory potency of the hyperthermophilic propeptide toward its cognate subtilase and its importance in regulating subtilase maturation, to prevent the degradation of the unprocessed subtilase precursors by the prematurely activated molecules.

**IMPORTANCE:**

Many microorganisms secrete proteases into their environment to degrade protein substrates for their growth. The important group of these extracellular enzymes are subtilases, which are also widely used in practical applications. These subtilases are inhibited by their propeptide domain, which is degraded during the prosubtilase maturation process. Here, we showed that the propeptide of pernisine, a prion-degrading subtilase from the hyperthermophilic archaeon, strongly inhibits pernisine with extraordinarily high binding affinity. This interaction proved to be highly selective, as pernisine propeptide was rapidly degraded by mesophilic pernisine homologs. This in turn allowed rapid transactivation of propernisine by mesophilic subtilases at lower temperatures, which might simplify the procedures for preparation of active pernisine for commercial use. The results reported in this study suggest that the hyperthermophilic subtilase propeptide evolved to function as tight and selective regulator of maturation of the associated prosubtilase to prevent its premature activation under high temperatures.

## INTRODUCTION

The subtilisin-like proteases, commonly referred to as subtilases, are a family of serine proteases that are widely distributed in eukaryotes, bacteria, and archaea, where they catalyze a number of cellular processes (1). Subtilases of microbial origin are mostly secreted into the extracellular environment, where they degrade proteins to provide nutrients or act as virulence factors (1, 2). The bacterial extracellular subtilases are also used commercially in different industries due to their high proteolytic activity and stability (3).

Inside the cells, the extracellular subtilases are generally synthesized as inactive zymogens (prosubtilases) consisting of the N-terminal signal peptide, the propeptide, and the subtilase (catalytic) domain (4, 5). The signal peptide directs the secretion of prosubtilase through the cell envelope (1), while the propeptide facilitates the folding and subsequent inhibition of the subtilase domain (6). Upon folding, the covalent complex of propeptide and subtilase is intramolecularly autoprocessed by autocatalytic cleavage of the scissile peptide bond between propeptide and subtilase to form the noncovalent complex (7, 8). Eventually, the propeptide dissociates from the complex and is autocatalytically degraded by the subtilase domain. This final and rate-limiting step of the prosubtilase maturation process leads to the formation of the active protease (9).

Pernisine is a highly thermostable extracellular subtilase produced by the hyperthermophilic archaeon *Aeropyrum pernix*. In addition to the well-characterized Tk-subtilisin (10) and pyrolysin (11), pernisine is another representative member of the hyperthermophilic subtilases that exhibit optimal activity at temperatures above 80°C. Mature pernisine retains most of its activity even after 12 h at 100°C (12). Due to the exceptional stability and prion-degrading activity of pernisine, this subtilase has high potential for practical applications, such as proteolytic decontamination of materials contaminated with infectious prion proteins and other resistant protein aggregates (13). Our previous studies showed that pernisine adopts the proteolytically active conformation independently of its propeptide (14), since the folding of pernisine catalytic domain is induced by Ca^2+^ ions (15). This is in contrast to subtilisins from *Bacillus* sp. which remain inactive when produced without their propeptides (16), as the chaperone activity of the propeptide is essential for the folding of the subtilisin domain (17). Similar to pernisine, Tk-subtilisin from the hyperthermophilic archaeon *Thermococcus kodakarensis* folds independently upon Ca^2+^ binding, whereas its propeptide only accelerates this process (18). Moreover, pernisine and Tk-subtilisin are effectively inhibited already at nanomolar concentrations of their propeptides (15, 19), whereas the mesophilic subtilisins from *Bacillus* sp. are completely inhibited only at micromolar propeptide concentrations (20). Apparently, the propeptides of hyperthermophilic extracellular subtilases act mainly as potent inhibitors of their cognate catalytic domains and to a lesser extent as chaperones. Remarkably, the mesophilic bacterial propeptides acquire ordered structure only in the presence of their cognate subtilisin domain (21), whereas propeptides from hyperthermophilic archaea fold independently (15, 22). The different folding pathways of these propeptides might be related to their inhibitory activity and also to their degree of selectivity toward subtilase inhibition.

Here, we used pernisine and its propeptide as a model for hyperthermophilic subtilase and propeptide domains to further investigate the inhibitory properties of the thermostable subtilase propeptide. We demonstrated that the ability of the pernisine propeptide to strongly inhibit its respective catalytic domain results in high selectivity of this interaction. Moreover, the pernisine propeptide and catalytic domain differed in their susceptibility to degradation by mesophilic subtilases. Consequently, we demonstrated that the tight inhibition of pernisine by its propeptide can be impaired by intermolecular propeptide degradation by mesophilic subtilases, allowing activation of propernisine at lower temperatures. Together with these analyses, we identified additional factors important for the regulation of propernisine maturation process and the final yield of active pernisine.

## RESULTS

### Pernisine propeptide does not inhibit or interact with pernisine homologs

**Fig 1:**
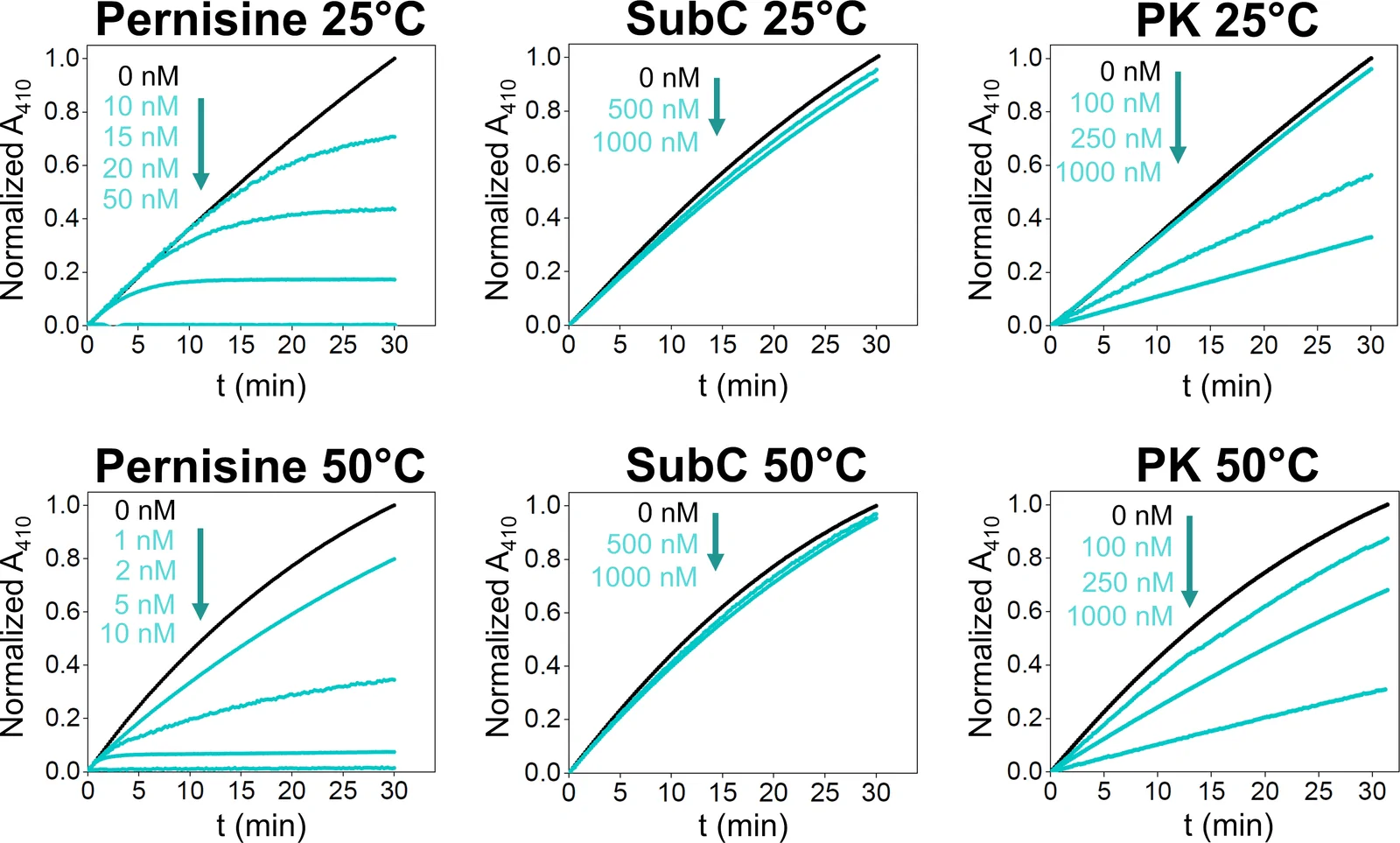
Inhibitory activity of pernisine propeptide against different subtilases. Hydrolysis of Suc-AAPF-*p*NA (0.3 mM) by pernisine (2 nM), SubC (1 nM), and PK (2 nM) was recorded in the absence (black lines) or presence of different concentrations of PRO^P^ (turquoise lines) at 25°C or 50°C, as indicated. PRO^P^ was mixed with Suc-AAPF-*p*NA before addition of the proteases, as described in Materials and Methods. The absorbance signal at 410 nm (*A*_410_) was normalized to the highest (final) *A*_410_ values of the reactions without PRO^P^. SubC, subtilisin Carlsberg; PK, proteinase K; PRO^P^, pernisine propeptide.

We compared the efficacy of pernisine propeptide (PRO^P^) in inhibiting pernisine and the two mesophilic subtilases homologous to pernisine, subtilisin Carlsberg (SubC) from *Bacillus licheniformis* and proteinase K (PK) from *Tritirachium album*. Although pernisine and its propeptide function optimally at approximately 90°C (15), the inhibition assays were conducted at lower temperatures (25°C and 50°C) to ensure the stability of SubC and PK during the experiments. PRO^P^ showed strong inhibition of its cognate protease pernisine at 25°C, with over 50% inhibition at 15 nM PRO^P^ and complete inhibition at 50 nM PRO^P^ (Fig. 1). The progress curves indicate slow-binding between PRO^P^ and pernisine. Consequently, inhibition of pernisine appeared to be stronger at elevated temperature (50°C), with inhibition exceeding 50% already at 2 nM PRO^P^ and complete inhibition at 5 nM PRO^P^. In contrast, pernisine homolog SubC was not inhibited even at 1,000 nM PRO^P^, either at 25°C or at 50°C. PK was only partially inhibited by PRO^P^ at severalfold higher PRO^P^ concentrations compared to pernisine inhibition. Complete inhibition of PK was not achieved even at 1,000 nM PRO^P^ at either temperature. Of note, the inhibition of PK by PRO^P^ did not occur through slow-binding, which indicates different mode of PK inhibition compared to pernisine.

**Fig 2:**
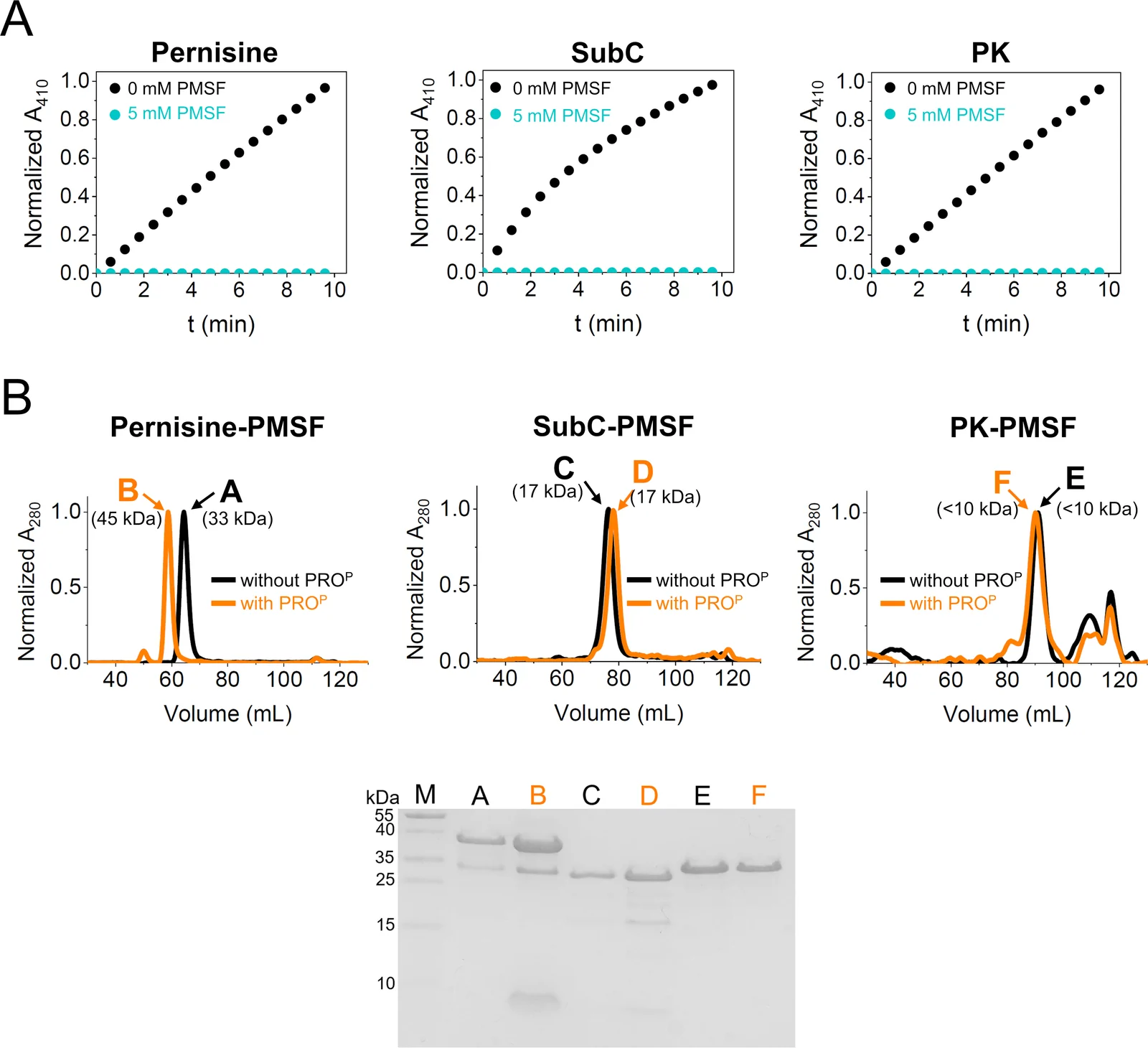
Analysis of complex formation between PRO^P^ and different subtilases. (**A**) Proteolytic activity of unmodified (black circles) or PMSF-modified (turquoise circles) subtilases (2 nM) pernisine, subtilisin Carlsberg (SubC), and proteinase K (PK), measured by Suc-AAPF-*p*NA (0.3 mM) hydrolysis at 25°C in 20 mM Tris (pH 8.0), 1 mM CaCl_2_. (**B**) SEC chromatograms of pernisine, SubC, and PK, either alone (black lines) or in the presence of PRO^P^ (orange lines) at room temperature. Apparent molecular weights are indicated above the corresponding peaks. Proteins from the SEC fractions corresponding to each chromatographic peak (**A–E**) were precipitated with trichloroacetic acid and resolved with Tricine-SDS-PAGE on 15% polyacrylamide gel. The chromatographic peaks and their corresponding SDS-PAGE lanes are labeled with the same letters. Black letters, subtilases without PRO^P^; orange letters, subtilases preincubated with PRO^P^ before SEC. Lane M, protein marker with molecular masses (kDa) indicated next to the gel. Pernisine-PMSF, pernisine inhibited by PMSF; SubC-PMSF, subtilisin Carlsberg inhibited by PMSF; PK-PMSF, proteinase K inhibited by PMSF; PRO^P^, pernisine propeptide.

Next, we characterized the interactions between PRO^P^ and pernisine, SubC or PK by size exclusion chromatography (SEC). To prevent degradation of PRO^P^ or subtilases during SEC analyses, all three proteases were pretreated with phenylmethylsulfonyl fluoride (PMSF), which completely abolished their proteolytic activity (Fig. 2A). Pernisine eluted from the SEC column as a single peak with an apparent molecular weight (*MW*_app_) of 33 kDa (Fig. 2B), which is in close agreement with the theoretical *MW* of His-tagged pernisine (35.4 kDa). When pernisine was preincubated with PRO^P^ before SEC analysis, the resulting peak shifted toward the lower elution volume, indicating the formation of a PRO^P^:pernisine complex (Fig. 2B). The *MW*_app_ of this complex (45 kDa) was consistent with its theoretical *MW* (43 kDa). Moreover, the fractions corresponding to this chromatographic peak contained both pernisine and PRO^P^ (Fig. 2B). Subtilases SubC and PK did not form stable complexes with PRO^P^, as their elution volume did not change in the presence of PRO^P^ (Fig. 2B). In addition, the peaks of SubC and PK did not contain PRO^P^ (Fig. 2B). The *MW*_app_ of SubC (17 kDa) and PK (less than 10 kDa) did not match their theoretical *MW* values (27.3 kDa SubC and 28.9 kDa PK). This could be due to the compactness of the conformation of these subtilases, which would increase their elution volume.

**Fig 3:**
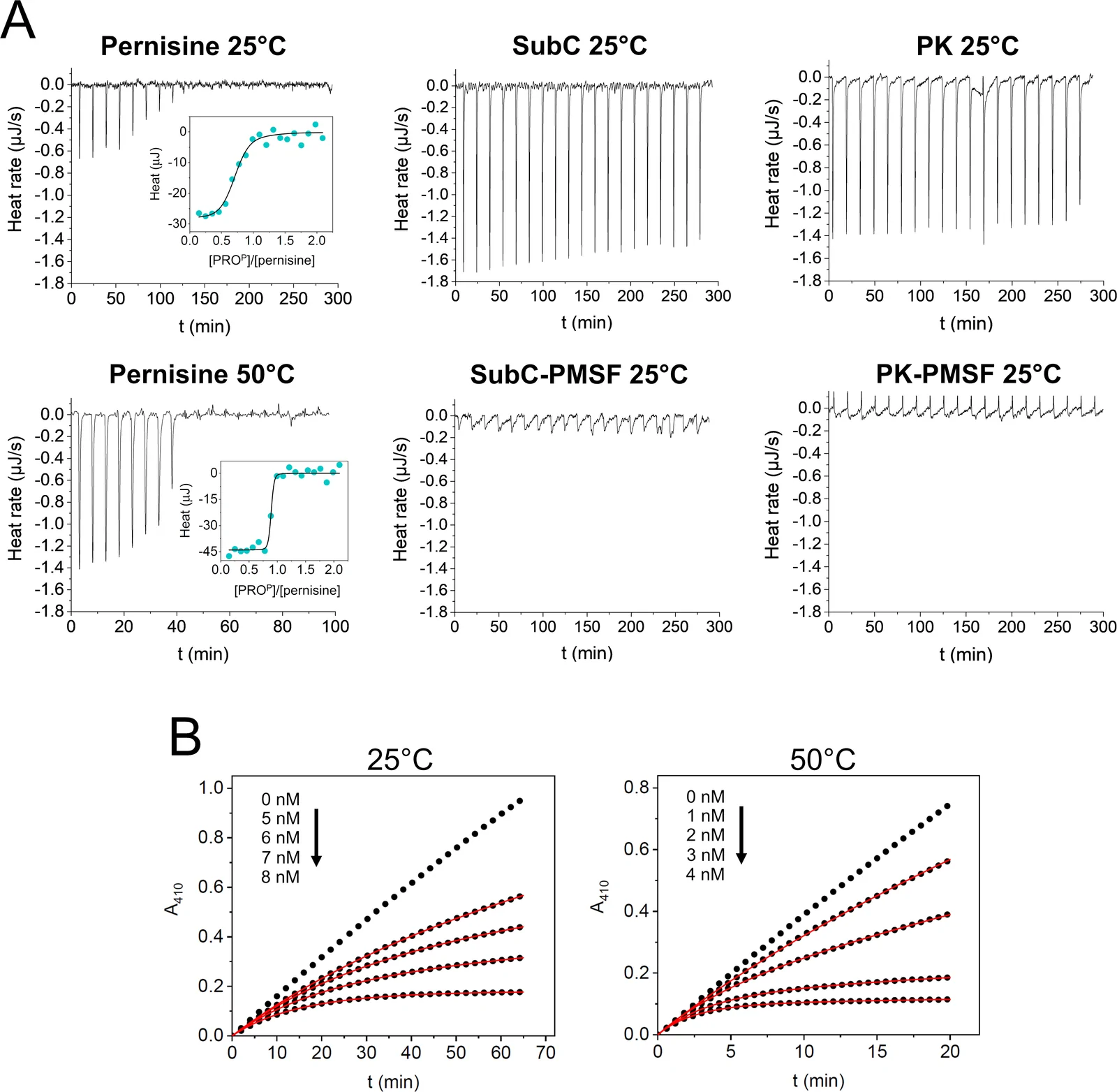
Analysis of interactions between PRO^P^ and different subtilases. (**A**) ITC thermograms of successive PRO^P^ injections into the solutions of pernisine, SubC, PK, and the PMSF-inhibited SubC and PK (SubC-PMSF and PK-PMSF, respectively). As indicated, ITC experiments were conducted at 25°C or 50°C. Integrated data from the pernisine titration with PRO^P^ are shown in the insets. SubC, subtilisin Carlsberg; PK, proteinase K. (**B**) Progress curves of Suc-AAPL-pNA hydrolysis by pernisine at different PRO^P^ concentrations at 25°C or 50°C, as indicated. Values of absorbance at 410 nm (*A*_410_) are shown as black dots, and fits to experimental data are shown as red lines. Data were fitted as described in the Materials and Methods.

We further investigated the interactions between PRO^P^ and the subtilases by isothermal titration calorimetry (ITC) (Fig. 3A). When PRO^P^ was injected into the sample cell containing either SubC or PK at 25°C, the heat changes resulted in exothermic peaks. The area of the corresponding peaks did not decrease significantly over the 19 injections of PRO^P^, which does not reflect the binding of PRO^P^ to SubC or PK. Apparently, these peaks resulted from rapid proteolytic degradation of PRO^P^ by SubC and PK. This could be inferred from the ITC experiment with PMSF-inhibited SubC or PK, in which no significant heat changes were observed after injections of PRO^P^. This highlights the lack of stable interactions between PRO^P^ and the two mesophilic subtilases. In contrast, the interactions between PRO^P^ and pernisine were evident at both 25°C and 50°C. The number of PRO^P^ molecules bound to pernisine (*n*) and the dissociation constant (*K*_D_) of the PRO^P^:pernisine complex were deduced from the corresponding titration curves (Fig. 3A). The values of *n* were 0.68 at 25°C and 0.89 at 50°C. Of note, the *K*_D_ at 25°C (96 ± 5 nM) was almost 100-fold higher than that at 50°C (1.3 ± 0.2 nM). The stronger interactions between PRO^P^ and pernisine at the higher temperature were also evident from the apparent inhibition constants (*K*_i_′) of the PRO^P^:pernisine complex. The *K*_i_′ was ~330 pM at 25°C and ~20 pM at 50°C, as derived from the Suc-AAPL-*p*NA hydrolysis progress curves (Fig. 3B). These *K*_i_′ values are significantly lower than the corresponding *K*_D_ values for the PRO^P^:pernisine complex, which can be attributed to the tight but slow-binding inhibition of pernisine by PRO^P^.

### Structural analysis of the interaction between propeptide and catalytic domain

**Fig 4:**
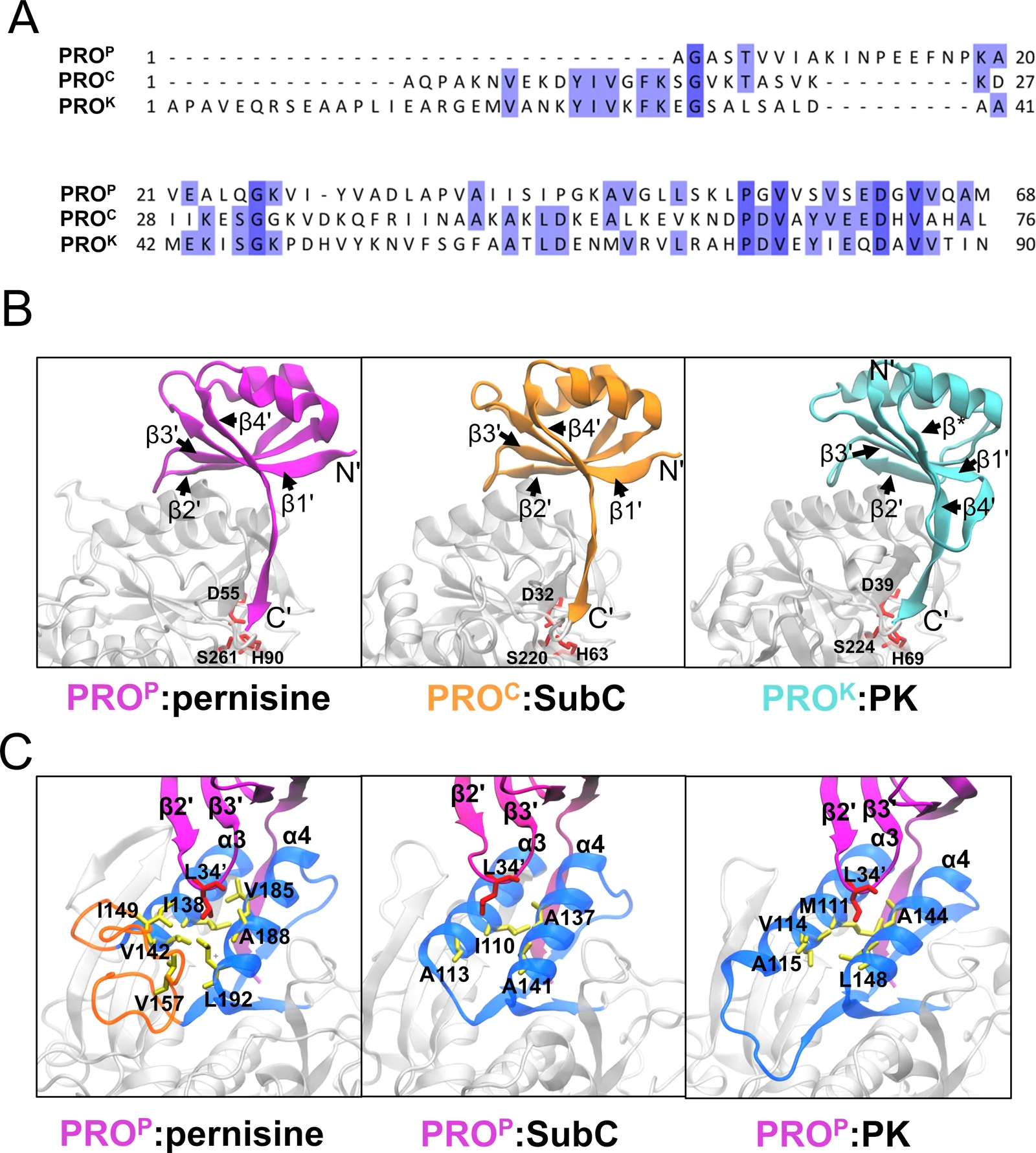
Structural analysis of PRO^P^ interaction with subtilases. (**A**) Comparison of amino acid sequences of pernisine, SubC, and PK propeptide domains (PRO^P^, PRO^C^, and PRO^K^, respectively). The sequences were aligned using Clustal Omega (25, 26) and visualized with Jalview (27). The amino acid residues that are conserved between all three sequences are shaded in darker blue. The residues conserved between only two sequences are shaded in lighter blue. Amino acid numbering is indicated at the end of each row. (**B**) 3D models of the propeptide domains of pernisine (magenta), SubC (orange), and PK (cyan) in complex with their corresponding catalytic domains (gray). The N′- and C′-termini of the propeptides are indicated. The arrows indicate the β-strands that form the β-sheet (β1–β4). The additional β-strand that is present only in PK is marked with β*. The side chains of the amino acid residues forming the Ser-His-Asp catalytic triad in the active site of the subtilase are shown as red sticks, with the corresponding residues named. (**C**) Interaction interface between PRO^P^ and different subtilases (pernisine, SubC, and PK, as indicated). PRO^P^ is shown in magenta, the two α-helices (α3 and α4) and the two β-strands of the “upper” part of the subtilase catalytic domain (gray) that form the interface with PRO^P^ are shown in blue. The unique loop between the α3 and downstream β-strand in pernisine is shown in orange. The amino acid residues on the subtilase domain that form the hydrophobic pocket for the protruding leucine of the propeptide (L34′; red sticks) are labeled and shown with yellow sticks. The two β-strands of PRO^P^ upstream and downstream of L34′ are labeled as β2′ and β3′, respectively, and correspond to the β2′- and β3′-strands of panel (B). Amino acid numbering begins with the N-terminus of PRO^P^ and N-termini of the catalytic domains of pernisine/SubC/PK. Models were generated using the ColabFold program (28) and visualized using the VMD software (29). PRO^P^, pernisine propeptide; PRO^C^, subtilisin Carlsberg propeptide; PRO^K^, proteinase K propeptide; SubC, subtilisin Carlsberg; PK, proteinase K.

To gain further insight into the observed specificity of PRO^P^ toward inhibition of pernisine, we applied the ColabFold implementation of Alphafold2-multimer to model the three-dimensional structures of the propeptide domains of pernisine, SubC, and PK (PRO^P^, PRO^C^, and PRO^K^, respectively) in complex with their cognate subtilases. The amino acid sequences of these three propeptides do not share any significant sequence similarity (Fig. 4A). Nevertheless, the modeled structures of PRO^P^ and PRO^C^ are highly similar and consist of a four-stranded antiparallel β-sheet positioned below the two α-helices (Fig. 4B). The modeled structure of PRO^K^ differs from that of PRO^P^ and PRO^C^ by an additional N-terminal strand that forms the β-sheet. In all modeled structures, the C-terminal extension of the propeptide sits in the active site cleft of the catalytic domain, which precludes the binding of substrate (Fig. 4B). This configuration is consistent with the published crystal structures of prosubtilisin BPN′ (PDB ID: 1SPB) (23), a close homolog of proSubC, and proTk-subtilisin (PDB ID: 2E1P) (24), a close homolog of propernisine. In addition, the structures of PRO^P^ in complex with the catalytic domains of pernisine, SubC, and PK were also modeled (Fig. 4C). PRO^P^ interacts with these catalytic domains via its β-sheet, which covers the region encompassing the α-helices 3 (α3) and 4 (α4) of the catalytic domain. In particular, Leu^34^′ in the loop between the β2′- and β3′-strands of PRO^P^ protrudes into the hydrophobic pocket on the catalytic domain. This pocket is formed by side chains of amino acid residues that constitute the α3, α4, and the β-strand between these two α-helices (Fig. 4C). Importantly, pernisine contains more hydrophobic amino acid residues in this region compared with SubC and PK. The long loop between the α3 helix and the following β-strand in pernisine contains Ile^149^ and Val^157^, whose side chains are oriented toward Leu^34^′ of PRO^P^ (Fig. 4C). Apparently, this loop additionally buries the hydrophobic Leu^34^′ of PRO^P^ and possibly contributes to the tight interaction between PRO^P^ and the catalytic domain of pernisine. The corresponding loop in SubC and PK is shorter and does not contain any hydrophobic amino acid residues oriented toward Leu^34^′ (Fig. 4C).

### Propeptide of pernisine is readily degraded by mesophilic subtilisins

**Fig 5:**
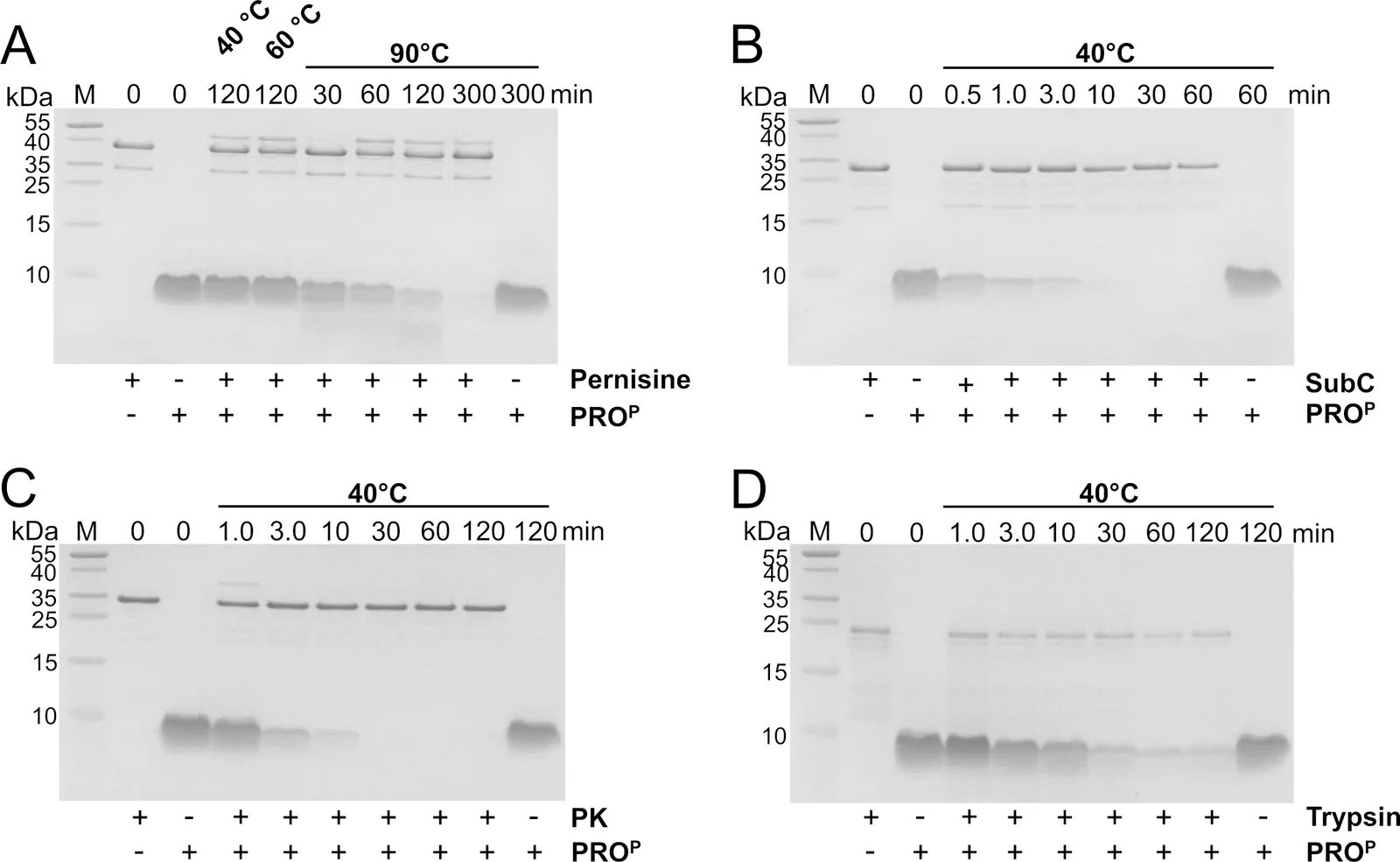
Degradation of free PRO^P^ by different proteases. PRO^P^ (25 µM) was incubated with (**A**) pernisine (2.5 µM), (**B**) SubC (2.5 µM), (**C**) PK (2.5 µM), and (**D**) trypsin (2.5 µM) at the indicated temperatures. Reactions were stopped at the indicated times with TCA and resolved by Tricine-SDS-PAGE on 15% polyacrylamide gels. The presence (+) or absence (−) of PRO^P^ and protease in each reaction is indicated below the gels. Lane M, protein markers with molecular masses (kDa) next to the gels. SubC, subtilisin Carlsberg; PK, proteinase K; PRO^P^, pernisine propeptide.

To investigate the susceptibility of PRO^P^ to proteolytic degradation, different subtilases (pernisine, SubC, and PK) were mixed with a 10-fold higher molar concentration of PRO^P^ and incubated at 40°C (SubC and PK) or 40°C–90°C (pernisine). The reaction products were resolved by SDS-PAGE. PRO^P^ was resistant to proteolysis by pernisine at 40°C and 60°C because the intensity of the ~7 kDa SDS-PAGE band, corresponding to PRO^P^, remained intact even after 120 min incubation with pernisine (Fig. 5A). Slow degradation of PRO^P^ by pernisine was observed at 90°C, with PRO^P^ still barely detectable after 5 h at this temperature (Fig. 5A). In contrast to pernisine, both SubC and PK completely hydrolyzed PRO^P^ after 30 min at 40°C. Noteworthy, the rate of PRO^P^ hydrolysis by SubC was higher compared to PK (compare Fig. 5B and C). PRO^P^ was also degraded by trypsin, a proteinase that is not of subtilase-type. However, this degradation was not completed after 120 min of incubation (Fig. 5D). Of note, when PRO^P^ was incubated with pernisine, an additional SDS-PAGE band of >40 kDa was observed at all incubation temperatures (Fig. 5A). This band indicates the formation of a covalent complex PRO^P^:pernisine upon interaction of these two domains. Similar observations were made after 1 min incubation of PRO^P^ with PK, where a weak band of ~37 kDa appeared, which could correspond to the covalent complex PRO^P^:PK (Fig. 5C).

**Fig 6:**
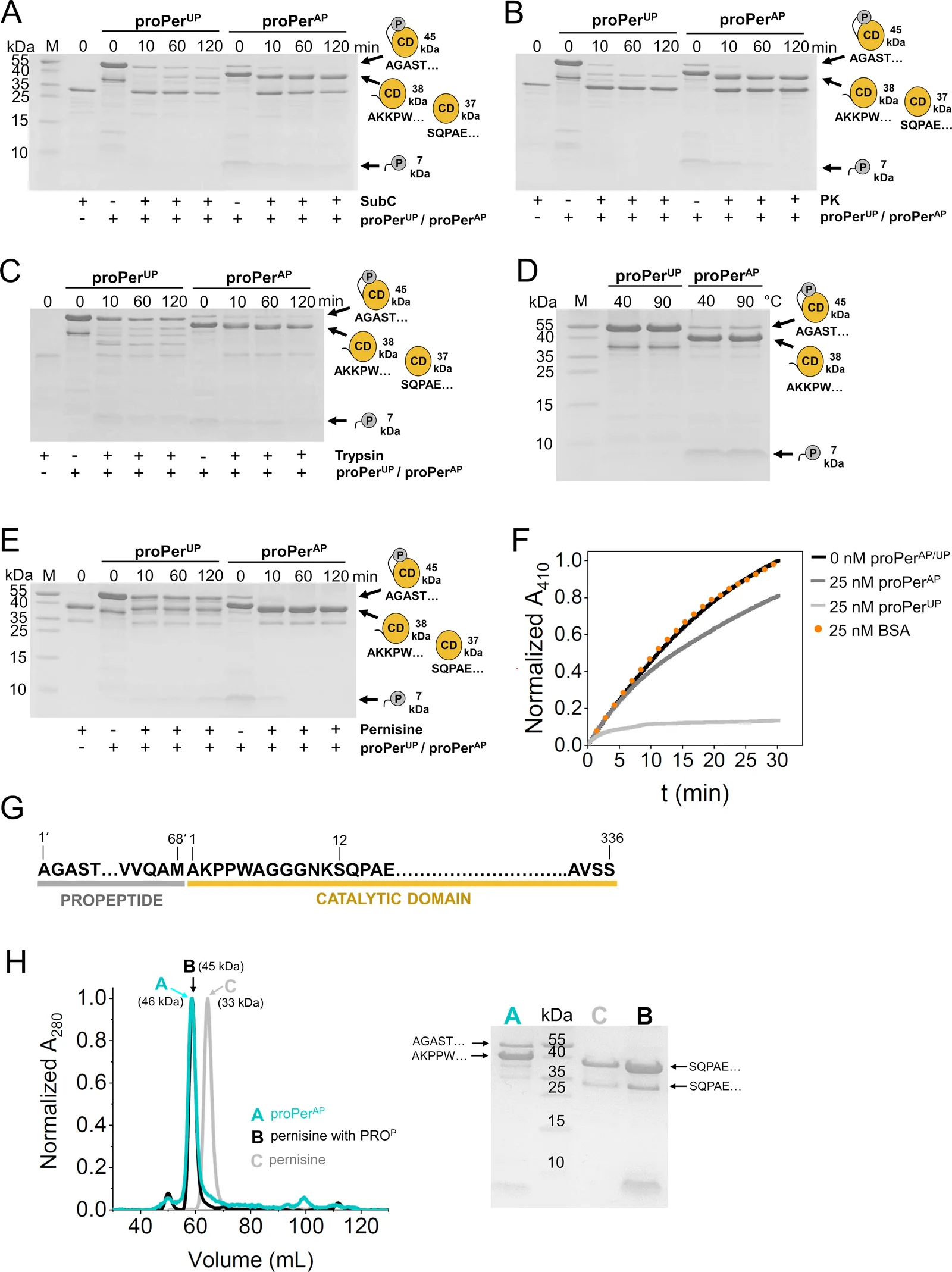
Degradation of PRO^P^ in complex with pernisine catalytic domain by different proteases. (**A−C**) The unprocessed and autoprocessed propernisine forms (proPer^UP^ and proPer^AP^, respectively) were incubated with (**A**) SubC, (**B**) PK, (**C**) trypsin, and (**E**) pernisine. The concentrations of each protein in the reactions were 0.8 µM. Reactions were incubated at 40°C (**A−C**) or 90°C (**E**), stopped at the indicated times with TCA and resolved by Tricine-SDS-PAGE on 15% polyacrylamide gels. The presence (+) or absence (−) of active protease and proPer^UP^ or proPer^AP^ in each reaction is indicated below the gels. (**D**) proPer^UP^ and proPer^AP^ incubated at 40°C or 90°C for 120 min without addition of the active proteases. Lane M, protein markers with molecular masses (kDa) next to the gels. The individual pernisine propeptide (P; gray) and catalytic (CD; yellow) domains, and their complex, are assigned to their corresponding SDS-PAGE bands with their respective molecular weights, as shown schematically next to the gels. The catalytic domain of pernisine is shown in both the untruncated (38 kDa) and truncated (37 kDa) forms. The N-termini indicated were determined by Edman degradation. (**F**) Activity of 2 nM pernisine was measured at 90°C, using the Suc-AAPF-pNA as substrate, in the absence (black line) or presence of 25 nM proPer^UP^ (light gray line) and 25 nM proPer^AP^ (dark gray line). Orange dots, activity of 2 nM pernisine in the presence of 25 nM BSA. The absorbance signal at 410 nm (*A*_410_) was normalized to the highest (final) *A*_410_ value of the control reaction that contained only pernisine and substrate. (**G**) N-terminal and C-terminal amino acid sequences of the propernisine domains. The sequences corresponding to PRO^P^ and pernisine catalytic domain are underlined with gray and yellow lines, respectively. The terminal amino acid residues of each domain and of Ser12 are numbered. (**H**) The chromatogram of proPer^AP^ (turquoise) is compared with chromatograms of pernisine (gray line) and pernisine mixed with pernisine propeptide (PRO^P^) (black line) that were analyzed with the same column. Proteins from the fractions corresponding to the different chromatographic peaks (A, proPer^AP^; B, pernisine with PRO^P^; C, pernisine) were precipitated with trichloroacetic acid and resolved by Tricine-SDS-PAGE on 15% polyacrylamide gel. The SDS-PAGE lanes are labeled with the same letters as the corresponding chromatographic peaks. The protein markers with the indicated molecular masses (kDa) are in the second lane from the left. The N-terminal amino acid sequences of the proteins in individual SDS-PAGE bands were determined by Edman degradation and are shown next to the gel. SEC and SDS-PAGE data from pernisine and pernisine-PRO^P^ samples used to visualize this panel are the same as in Fig. 2B. SubC, subtilisin Carlsberg; PK, proteinase K; proPer^UP^, unprocessed propernisine; proPer^AP^, autoprocessed propernisine; BSA, bovine serum albumin.

Next, we investigated whether different proteases hydrolyze PRO^P^ even when this propeptide is complexed with its cognate catalytic domain (pernisine). For this purpose, we used either unprocessed or autoprocessed propernisine as substrate. The unprocessed propernisine (proPer^UP^) is a covalent complex PRO^P^:pernisine, with the scissile peptide bond Met^68^′-Ala^1^ between these two domains intact. To produce proPer^UP^, the catalytic Ser^261^ in the pernisine domain was replaced with Ala. This mutation completely abolishes the proteolytic activity of the pernisine domain and thus prevents the autoprocessing and further maturation of propernisine. The unprocessed state of proPer^UP^ was verified by SDS-PAGE, where it migrated as a ~45 kDa band (Fig. 6A through E). The N-terminal sequence of the protein from this ~45 kDa band was determined to be Ala^1^′-Gly^2^′-Ala^3^′-Ser^4^′-Thr^5^′, which corresponds to the N-terminus of PRO^P^ (Fig. 6G). The autoprocessed propernisine (proPer^AP^) is the noncovalent complex PRO^P^:pernisine, with cleaved peptide bond Met^68^′^-^Ala^1^. This was achieved by replacing the catalytic Ser^261^ with Cys, which allowed autoprocessing of propernisine (i.e., autocleavage of the peptide bond Met^68^′-Ala^1^), but not further propernisine maturation by degradation of PRO^P^. The autoprocessed state of proPer^AP^ was verified by SEC, where proPer^AP^ migrated as a ~46 kDa protein, but dissociated into PRO^P^ (~7 kDa band) and the catalytic domain of pernisine (~38 kDa band) in SDS-PAGE (Fig. 6H). The N-terminal sequence of the protein from this ~38 kDa SDS-PAGE band was determined to be Ala^1^-Lys^2^-Pro^3^-Pro^4^-Trp^5^, which corresponds to the N-terminus of the catalytic domain. This confirms that the scissile peptide bond that is cleaved during propernisine autoprocessing is located at Met^68^′-Ala^1^ (Fig. 6G and H).

The proPer^UP^ and proPer^AP^ molecules were incubated with different subtilases (SubC/PK/pernisine) or trypsin, and the reaction products were analyzed by SDS-PAGE. The majority of proPer^UP^ molecules (~45 kDa SDS-PAGE band) were degraded within 10 min when incubated with SubC (Fig. 6A) or PK (Fig. 6B) at 40°C. Upon further incubation, some of the remaining proPer^UP^ molecules were converted to the truncated form (~37 kDa SDS-PAGE band) (Fig. 6A and B). N-terminal sequencing confirmed that this truncated form corresponds to the catalytic domain of pernisine with the N-terminus Ser^12^-Gln^13^-Pro^14^-Ala^15^-Glu^16^. Therefore, SubC and PK degraded the PRO^P^ domain of proPer^UP^, along with the 11 N-terminal amino acid residues of pernisine catalytic domain. This truncation of the catalytic domain also occurs during the automaturation of propernisine (15).

The autoprocessed proPer^AP^ dissociated into the ~38 kDa catalytic domain and ~7 kDa PRO^P^ on SDS-PAGE. Both SubC and PK degraded the PRO^P^ domain of proPer^AP^ during incubation at 40°C (Fig. 6A and B). In contrast, the catalytic domain of proPer^AP^ was resistant to degradation and was only truncated by ~1 kDa after 10 min of incubation (Fig. 6A and B). The resulting ~37 kDa SDS-PAGE band corresponded to the catalytic domain of pernisine truncated by 11 N-terminal residues as described above. Of note, a portion of the molecules in the proPer^AP^ samples migrated at ~45 kDa (see Fig. 6A and B, lanes with proPer^AP^ at 0 min), as autoprocessing of propernisine^S261C^ (described in Materials and Methods) was incomplete, even after 2 h at 100°C. This remaining unprocessed form was degraded by both SubC and PK within 60 min.

Trypsin degraded the unprocessed proPer^UP^, whereas both the catalytic and PRO^P^ domains of the autoprocessed proPer^AP^ were resistant to hydrolysis by this protease (Fig. 6C). In the absence of the active proteases, proPer^UP^ and proPer^AP^ did not undergo any proteolytic transformations, as their SDS-PAGE profiles remained the same after 120 min at 40°C or 90°C (Fig. 6D).

The mature pernisine that was added *in trans* degraded a some of the proPer^UP^ molecules after 10 min at 90°C (Fig. 6E). Notably, this mature pernisine hydrolyzed the catalytic domain of proPer^UP^, leaving the PRO^P^ domain intact, as indicated by the presence of the ~7 kDa band. Note that the ~37 kDa bands in Fig. 6E correspond to the mature pernisine added *in trans* and not the catalytic domain of proPer^UP^. The remaining proPer^UP^ molecules were not further degraded with continued incubation, as the intensity of the ~45 kDa band remained the same after 60 min and 120 min (Fig. 6E). Importantly, the proPer^UP^ was degraded to a lesser extent by the mature pernisine (Fig. 6E) than by SubC (Fig. 6A), PK (Fig. 6B), or trypsin (Fig. 6C). Apparently, PRO^P^, which was released after degradation of proPer^UP^ catalytic domain, inhibited mature pernisine. In contrast, mature pernisine completely degraded the PRO^P^ domain of autoprocessed proPer^AP^, whereas its catalytic domain was only truncated by ~1 kDa (Fig. 6E). The resistance of proPer^AP^ catalytic domain to proteolysis likely rendered its associated PRO^P^ domain inaccessible for inhibition of mature pernisine and enabled the degradation of PRO^P^ by this mature pernisine. These observations are confirmed by real-time measurements of the proteolytic activity of pernisine, where pernisine was more strongly inhibited by proPer^UP^ than by proPer^AP^ (Fig. 6F).

### Propernisine is stabilized by its autoprocessing

**Fig 7:**
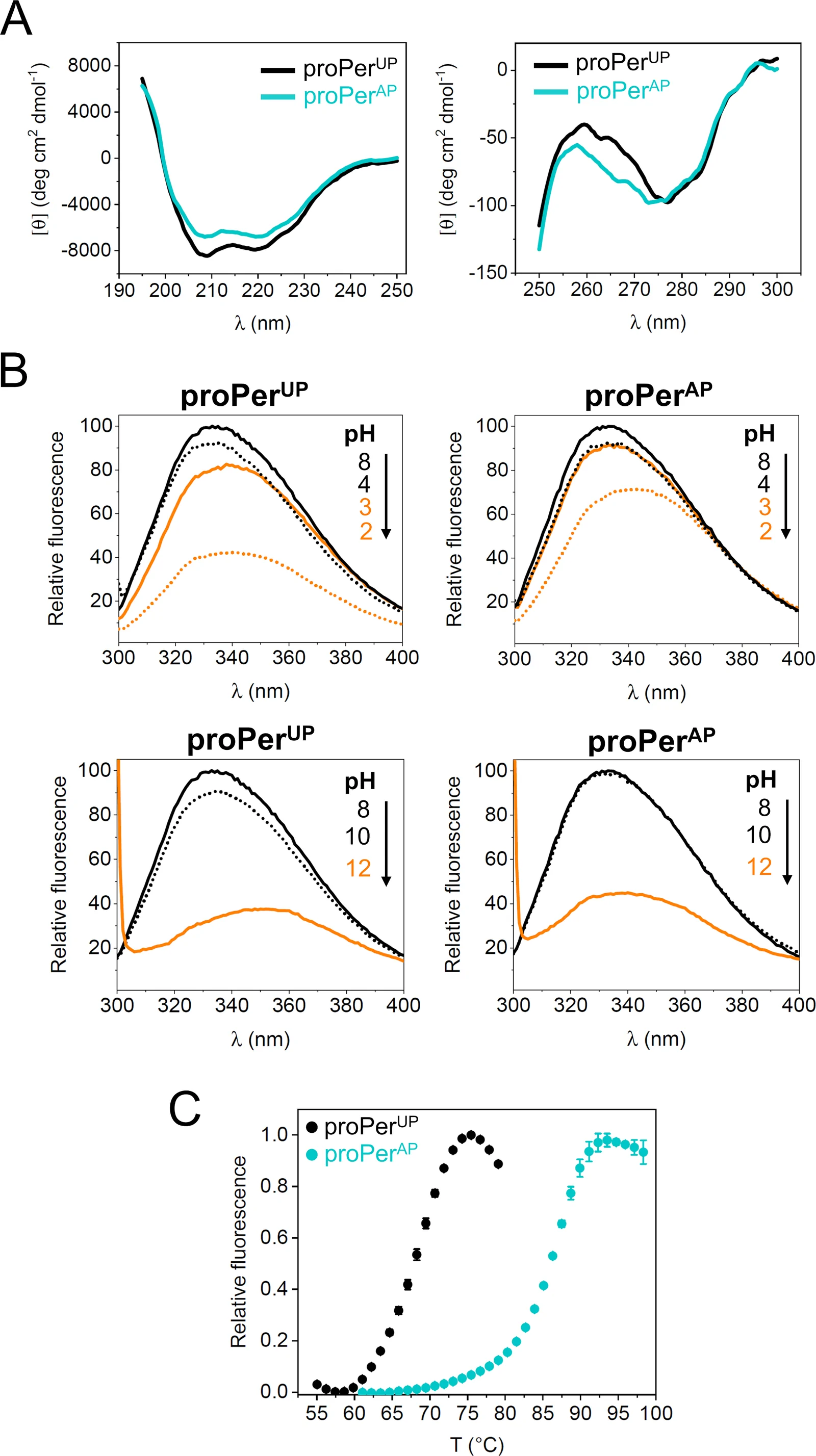
Conformational stabilities of unprocessed and autoprocessed propernisine. (**A**) The far-UV (left panel) and near-UV (right panel) CD spectra. Black lines, proPer^UP^; turquoise lines, proPer^AP^. The CD spectra were recorded in 10 mM Tris (pH 8.0), 10 mM CaCl_2_ at 20°C. (**B**) Intrinsic fluorescence spectra of proPer^UP^ and proPer^AP^ at different pH values, as indicated on the diagrams. Top diagrams: black solid line, pH 8; black dotted line, pH 4; orange solid line, pH 3; orange dotted line, pH 2. Bottom diagrams: black solid line, pH 8; black dotted line, pH 10; orange solid line, pH 12. (**C**) Differential scanning fluorimetry of the proPer^UP^ (black dots) and proPer^AP^ (turquoise dots) in 50 mM citrate buffer (pH 5.0), 2.5 mM CaCl_2_. SYPRO Orange dye was used as the fluorophore, as described in Materials and Methods. Data are means ± standard deviation of three replicates. proPer^UP^, unprocessed propernisine; proPer^AP^, autoprocessed propernisine.

The results described above suggest that unprocessed propernisine is less proteolytically stable than its autoprocessed form. We investigated whether these different proteolytic stabilities are also reflected in structural differences between the two propernisine states. The far-UV CD signal indicated similar α-helical secondary structures of proPer^UP^ and proPer^AP^, with a slightly weaker CD-signal of proPer^AP^ compared with proPer^UP^ (Fig. 7A, left panel). The near-UV spectrum of proPer^UP^ showed a strong band at ~280 nm (Fig. 7A, right panel), which is contributed by tyrosine residues (30). This band was shifted by 5 nm toward shorter wavelengths in the spectrum of proPer^AP^, suggesting a slight change in the tertiary structure of propernisine during autoprocessing.

In addition, we investigated whether the higher proteolytic stability of autoprocessed propernisine was also related to its conformational stability. Since temperatures up to 100°C are not sufficient for destabilization of propernisine at physiological pH values (15), the conformational stabilities of proPer^UP^ and proPer^AP^ were first investigated using the intrinsic tryptophan fluorescence emission measured at different pH values (Fig. 7B). The fluorescence intensity of proPer^UP^ decreased and its emission maximum shifted to longer wavelengths at lower pH values. This indicates an increased exposure of the tryptophan residues to the solvent. Similar observations were made for proPer^AP^ when the pH was lowered, but its fluorescence intensity decreased to a lesser extent compared to proPer^UP^. The autoprocessed propernisine form also appeared to be more stable than the unprocessed form under alkaline conditions. At pH 10, the fluorescence intensity of proPer^UP^ decreased by ~10% compared to that at pH 8, while the fluorescence of proPer^AP^ remained unchanged. The fluorescence intensities of both proteins were significantly reduced at pH 12. Notably, the red shift of the emission maximum at pH 12 was more pronounced for proPer^UP^ (*λ*_max_ = 350 nm) than for proPer^AP^ (*λ*_max_ = 340 nm). These results demonstrate the higher conformational stability of the autoprocessed propernisine form. This is also evident from the melting profiles of proPer^UP^ and proPer^AP^ measured by differential scanning fluorimetry (DSF) at pH 5, since this pH was sufficient for the initial destabilization of proPer^UP^ and proPer^AP^. The transition to the unfolded state upon heating was evident for both proteins (Fig. 7C), and the half-point of this transition was defined as the melting temperature (*T*_m_). The *T*_m_ of proPer^UP^ (67.8 ± 0.2°C) was significantly lower than the *T*_m_ of proPer^AP^ (85.2 ±0.4°C), which underlines the higher conformational stability of the autoprocessed propernisine form.

### Mesophilic subtilases enable *in trans* propernisine maturation at lower temperatures

**Fig 8:**
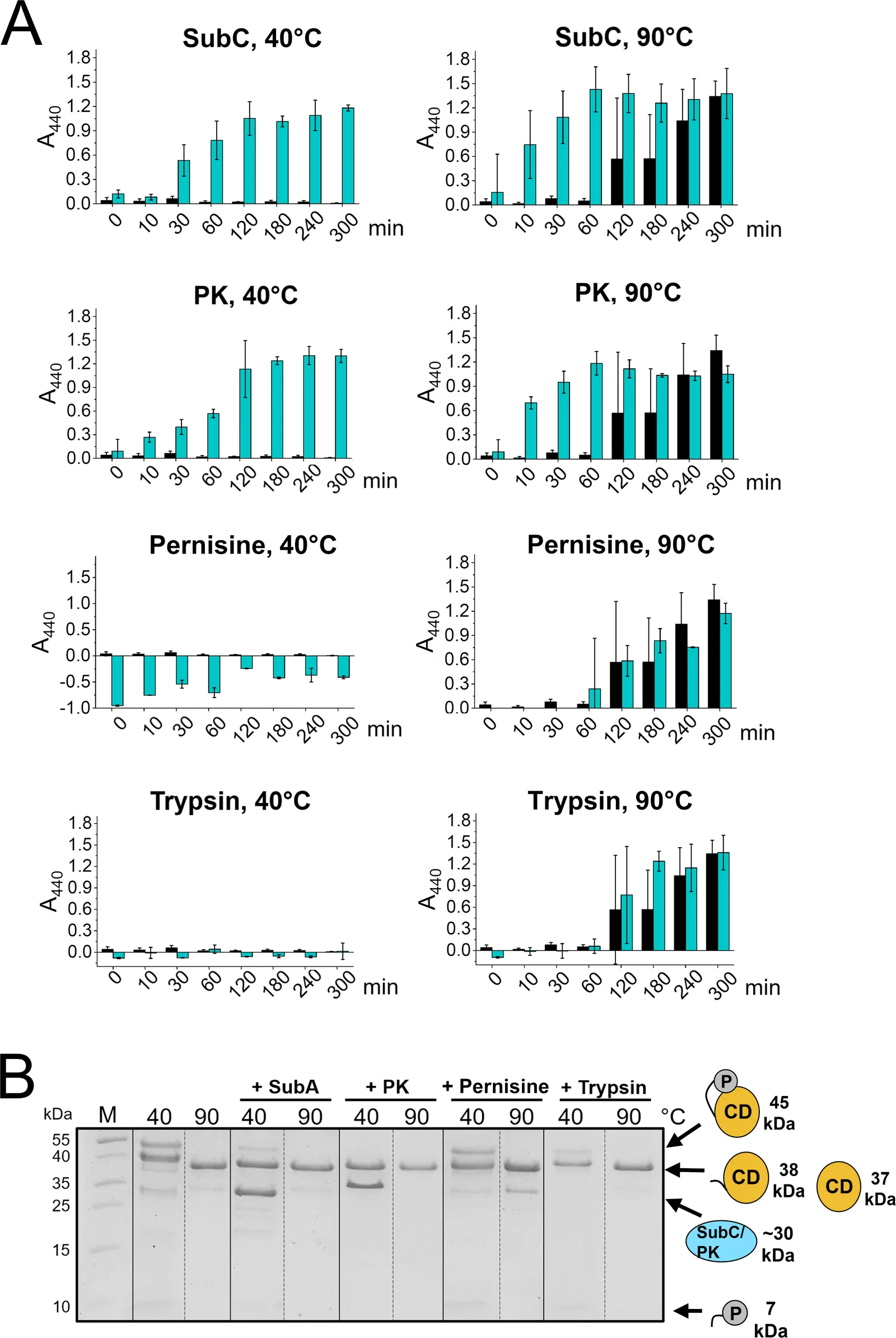
Transactivation of propernisine by different proteases. (**A**) Proteolytic activities of pernisine after its preincubation without (black bars) or with (turquoise bars) the active proteases (SubC, PK, pernisine, or trypsin) at 40°C or 90°C, as indicated. The concentrations of individual proteins in the preincubation reactions were 1 µM. At the indicated time points, the preincubation reactions were frozen at −20°C until their proteolytic activities were determined. Of note, the reactions that were preincubated at 40°C and contained SubC, PK, or trypsin were exposed to 90°C for 60 s before freezing to denature these mesophilic proteases. Proteolytic activities in each preincubated sample were determined at 90°C using azocasein as substrate, as described in the Materials and Methods. Error bars are standard deviations of the three independently preincubated samples. (**B**) Tricine-SDS-PAGE analysis of propernisine after 300 min incubation without or with the active proteases (SubC, PK, pernisine, or trypsin) at 40°C or 90°C, as indicated above the gel. The proteases SubC, PK, the individual pernisine propeptide (P; gray), and catalytic (CD; yellow) domains, and their complex, are assigned to the corresponding SDS-PAGE bands with their respective molecular weights shown schematically next to the gels. Lane M, protein markers with molecular masses (kDa) shown next to the gels. SubC, subtilisin Carlsberg; PK, proteinase K.

The results in Fig. 5 and 6 show that the PRO^P^ domain of propernisine can be degraded at mesophilic temperatures by SubC and PK, either when PRO^P^ is free or complexed with pernisine catalytic domain. This prompted us to investigate whether SubC and PK are able to activate propernisine *in trans*. Propernisine was incubated either alone or with different proteases before determining the proteolytic activity of pernisine with azocasein as substrate (Fig. 8A). At 40°C, propernisine alone was not autoactivated during the 5 h of incubation. When SubC or PK was added, the proteolytic activity of pernisine was detected after 30 min and 10 min, respectively, and maximum pernisine activity was reached after 2 h of incubation. At 90°C, propernisine underwent autoactivation and reached full activity after 4 h. However, in the presence of SubC or PK, pernisine was fully activated after 1 h. Therefore, both SubC and PK activated propernisine *in trans* also at 90°C.

In contrast to SubC and PK, mature pernisine did not transactivate propernisine at 40°C (Fig. 8A). Instead, pernisine was inhibited by propernisine at this temperature. This is evident from the negative absorbance values at 440 nm (A_440_) after subtraction of the A_440_ values of the control samples in which only pernisine (without propernisine) was present. This inhibition was not observed at 90°C, where the activation rate of propernisine was not affected by the addition of mature pernisine. Moreover, trypsin was not able to transactivate propernisine at either temperature, as no pernisine activity was observed during the 5 h incubation at 40°C and the rate of increase of A_440_ was similar at 90°C with or without trypsin (Fig. 8A). This is consistent with the observed inability of trypsin to efficiently degrade PRO^P^ (Fig. 5D).

Reaction samples from the transactivation experiments were analyzed with SDS-PAGE after the 5 h incubation at 40°C or 90°C (Fig. 8B). Propernisine that was incubated alone at 40°C migrated as the ~45 kDa band (unprocessed form) and ~38 kDa band (catalytic domain), accompanied by the ~7 kDa band (PRO^P^). Thus, the propernisine sample consisted of both the unprocessed and autoprocessed inactive forms, which are obtained after the purification from *Escherichia coli* (15). After incubation at 90°C, only the mature pernisine was present (~37 kDa band) (Fig. 8B), which was due to the autoactivation of propernisine under these conditions (Fig. 8A). This mature pernisine form was also seen after incubation in the presence of SubC or PK at 40°C or 90°C (Fig. 7B), which is due to transactivation of propernisine by these two proteases (Fig. 8A). Of note, SubC and PK were degraded by mature pernisine at 90°C (Fig. 8B). In the presence of mature pernisine or trypsin added *in trans*, propernisine was converted to the mature form only at 90°C. As described above (Fig. 8A), this mature form likely resulted from propernisine autoactivation under these conditions and not from transactivation by the proteases added *in trans*.

## DISCUSSION

The propeptide of pernisine (PRO^P^) acts as a tight, slow-binding inhibitor of pernisine, as shown by enzyme kinetic experiments (Fig. 3B). A similar slow-binding mode of inhibition by cognate propeptides was also reported for mesophilic subtilisins (20, 31), hyperthermostable Tk-subtilisin (19), and cathepsins (32, 33). The dissociation and inhibition constants determined for the PRO^P^:pernisine complex at 25°C (*K*_D_ ≈ 100 nM, *K*_*i*_′ ≈ 0.3 nM) in the present study (Fig. 3A and B) were severalfold lower than the corresponding constants reported for mesophilic subtilisins and their propeptides with *K*_D_ around 1 µM (34) and *K*_*i*_ from 1 nM to 88 nM (20). This indicates a tighter binding between PRO^P^ and pernisine already at 25°C, but the association of the PRO^P^:pernisine complex seems to be even stronger at the higher temperature (50°C), as indicated by the *K*_D_ value of 1.3 nM. This is similar to the *K*_D_ of Tk-subtilisin and its cognate propeptide (1.4 nM) determined at 40°C (35). Remarkably, the *K*_*i*_ of PRO^P^:pernisine at 50°C was in the picomolar range (~20 pM), which makes PRO^P^ an exceptionally potent biological inhibitor of pernisine. The strong inhibition of pernisine by PRO^P^ was also indicated by an apparent covalent complex observed after incubation of pernisine with PRO^P^ (Fig. 5A, >40 kDa SDS-PAGE band). Previously, the re-ligation of the peptide bond between the C-terminus of the propeptide and the N-terminus of the protease domain was reported for the subtilase of *Bacillus* sp. WF146 (36) and kumamolisin-As mutant (37). Since the N-terminus of pernisine is truncated after maturation (15), it is unlikely to be available for re-ligation with the C-terminal residue of PRO^P^. Speculatively, the observed covalent PRO^P^:pernisine complex might correspond to an acyl-enzyme intermediate resulting from a nucleophilic attack of catalytic Ser^261^ on the C-terminal Met^68^′ of PRO^P^, similar to that shown for the protein protease inhibitor bound to subtilisin BPN′ (38, 39).

Despite the strong interaction between PRO^P^ and its cognate protease pernisine, PRO^P^ was unable to inhibit and form stable complexes with the mesophilic pernisine homologs SubC and PK (Fig. 1; Fig. 2B and Fig. 3A). Therefore, the interaction between PRO^P^ and pernisine appears to be selective. This selectivity was also reported for tomato subtilase 3, which was effectively inhibited by its cognate propeptide but not by propeptides from other related plant subtilases (40). However, the observed selectivity of PRO^P^ contrasts with various subtilisin propeptides of *Bacillus* sp., which can inhibit heterogeneous subtilisins despite the significant differences in the amino acid sequences of these propeptides (20, 31, 41, 42). Since these propeptides of mesophilic bacterial subtilisins are disordered in isolated form and acquire their structure only in the presence of the subtilisin domain (43, 44), binding of propeptides to these subtilisins might require sufficient flexibility in the propeptide to adopt a conformation suitable for stable interaction. This could partly explain the inability of PRO^P^ to interact with and inhibit mesophilic subtilisins, since PRO^P^ is already folded in its free form (15). Moreover, the tighter association between PRO^P^ and pernisine might be also due to the greater hydrophobicity at the interaction interface in pernisine compared to SubC and PK, as shown by the structural models of these subtilases (Fig. 4C). Noteworthy, the additional hydrophobic side chains in pernisine are contributed by the long surface loop that is formed on account of the insertion sequence in pernisine catalytic domain. This surface loop also presumably forms four Ca^2+^-binding sites that mediate the folding of pernisine (15), which is inferred from the structural studies of the hyperthermostable Tk-subtilisin with similar insertion (24, 45).

In our previous study, we showed that the tertiary structure of free PRO^P^ is destabilized only at 90°C. Here, we showed that pernisine degrades PRO^P^ at 90°C but not at 60°C or 40°C (Fig. 5A). This suggests that destabilization of PRO^P^ at 90°C leads to its susceptibility to degradation by pernisine, which explains the need for high temperatures for complete maturation of propernisine (15). In contrast to PRO^P^, the isolated propeptides of the mesophilic subtilases of *Bacillus* sp. are structurally disordered, which makes them susceptible to degradation by their cognate subtilisin domains even at lower temperatures (9). Several studies showed that the propeptide variants that can fold in the absence of their cognate subtilase are more potent subtilisin inhibitors due to their higher intrinsic stability and apparent resistance to hydrolysis by subtilisin (19, 41, 46 – 48). However, the folded and thermostable PRO^P^ was readily degraded by the mesophilic subtilases SubC and PK at lower temperature (40°C) (Fig. 5B and C). This suggests that the linkage between the conformational and proteolytic stability of subtilase propeptides is not definite. Accordingly, Daugherty et al. (49) designed thermostable propeptide variants that acquired secondary structure independently but were weaker subtilisin inhibitors than the wild-type propeptide. It could be argued that the inhibitory potency and proteolytic stability of a propeptide are determined by its C-terminal tail, which binds into the active site cleft of the subtilase domain in a product-like manner (23, 24). This C-terminal tail contributes to, but is not essential for, inhibition of the eukaryotic subtilases cucumisin and PfSUB by their propeptides (50 – 52). However, Uehara et al. (53) reported that the C-terminal residue of the Tk-subtilisin propeptide affects its inhibitory potency. Similarly, replacement of the six C-terminal amino acids in the fungal protease inhibitors YIB2 and POIA1 with those of the propeptide of subtilisin BPN′ significantly increased their inhibitory potency toward subtilisin BPN′ (54, 55). Moreover, subtilisin BPN′ propeptide was shown to contain specific cleavage sites on its surface that are preferentially targeted by its cognate protease during propeptide degradation (56). In light of these studies, it is likely that PRO^P^ has evolved to tightly and selectively inhibit pernisine not only by stabilizing its overall structure but also by optimizing its C-terminal region and accessible amino acid segments on the PRO^P^ surface. The combination of these factors apparently prevents the degradation of PRO^P^ and the premature autoactivation of propernisine after its synthesis in a high-temperature environment.

PRO^P^ was degraded by SubC and PK, even when this propeptide was complexed with pernisine (Fig. 6A and B). This allowed transprocessing of the inactive PRO^P^:pernisine complex (propernisine) by SubC and PK into the active pernisine. Transactivation by heterogeneous proteases was also previously described for the thermostable subtilisin WF146 (36). Importantly, transactivation of propernisine by SubC and PK occurred at 40°C (Fig. 8A), whereas propernisine alone is autoactivated only at temperatures above 80°C (15). The susceptibility of propernisine to transactivation by the mesophilic subtilisin-like proteases has no physiological significance because these proteases do not coexist in nature. However, it is possible that another hyperthermophilic protease present in the marine habitat of *A. pernix*, the producer of pernisine, accelerates the activation of secreted propernisine *in situ*. In this context, it is noteworthy that mature pernisine did not significantly increase the rate of propernisine maturation *in trans* (Fig. 8A). This is in contrast to the maturation of prosubtilisin from *Bacillus* sp., where the active subtilisin molecules formed earlier in the maturation process then transactivate the remaining prosubtilisin molecules, which accelerates the overall maturation (34).

Together with the PRO^P^ degradation analyses, we found that the unprocessed propernisine was proteolytically unstable, whereas the autoprocessed form was resistant to degradation by proteases added *in trans* (Fig. 6). The unprocessed form appeared less stable despite the presence of Ca^2+^ ions that induce folding of the unprocessed propernisine into the ordered conformation (15). Moreover, the autoprocessed propernisine was also more conformationally stable than the unprocessed form (Fig. 7B and C). The increase in proteolytic and conformational stability after propernisine autoprocessing could be due to subtle structural changes, as indicated by the CD analyses (Fig. 7A). For instance, proTk-subtilisin forms one of its thermostabilizing Ca^2+^-binding sites after its autoprocessing, without any significant change in the overall structure of proTk-subtilisin (45, 57). The corresponding Ca^2+^-binding site was also found in the pernisine sequence (15). However, since proTk-subtilisin is resistant to proteolytic degradation even before autoprocessing (24), further structural changes might occur during autoprocessing of propernisine to increase its proteolytic stability during the subsequent steps of the maturation process.

**Fig 9:**
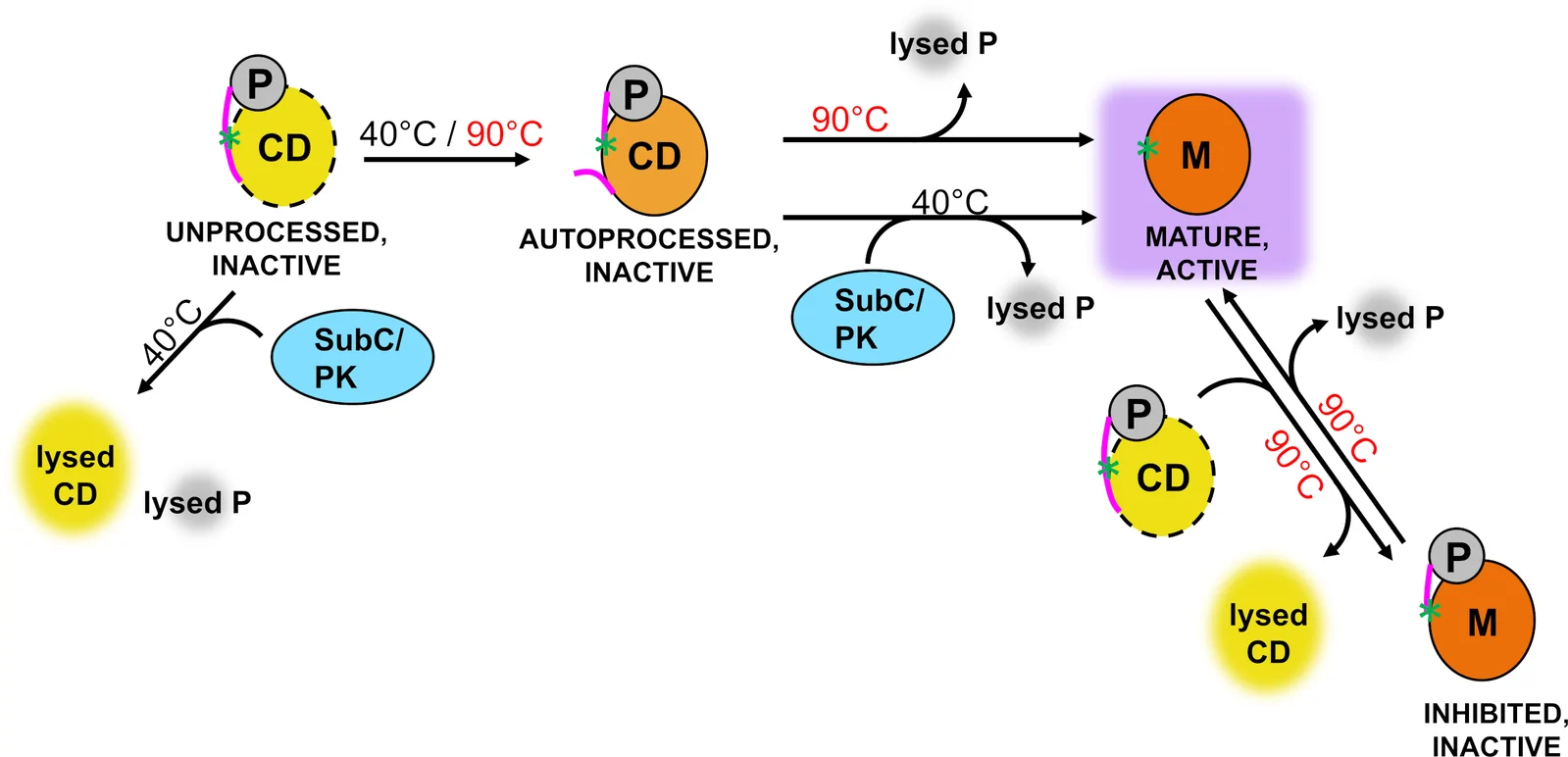
Schematic representation of propernisine activation pathways. The catalytic domain of unprocessed propernisine (CD; shown in yellow, outlined in dashed line) is proteolytically unstable and can be degraded at low temperatures (40°C) by mesophilic subtilases (SubC and PK; shown in blue). The propeptide domain of unprocessed propernisine (P; shown in gray) is also hydrolyzed by SubC and PK. In the absence of SubC or PK, propernisine undergoes autoprocessing (i.e., cleavage of the peptide linker between the catalytic domain and the propeptide shown in magenta) at either low (40°C) or high (90°C) temperature. After autoprocessing, the catalytic domain is stabilized (shown in orange, outlined by a solid line). The autoprocessed propernisine can then be converted to the active, mature pernisine (M; shown in dark orange, outlined by a solid line) either by autocatalytic degradation of its own propeptide at high temperature (90°C) or by transactivation by SubC or PK at low temperature (40°C). The mature pernisine can be reversibly inhibited by the propeptide that is released from the unprocessed propernisine after the mature pernisine-mediated degradation of the unstable propernisine catalytic domain.

Taken together, the selective behavior of the hyperthermophilic propeptide is associated with its high susceptibility to degradation by homologous subtilases. In contrast, the catalytic domain of pernisine becomes resistant to proteolysis after stabilizing its conformation upon autoprocessing. This, in turn, allows preparation of the mature pernisine from its inactive proform by transactivation with mesophilic subtilases at lower temperatures and faster rates compared to the autocatalytic maturation of propernisine. These findings are summarized graphically in Fig. 9. Furthermore, stabilization of the catalytic domain after autoprocessing apparently prevents its degradation by the mature pernisine molecules that are formed earlier in the maturation process. However, the mature pernisine was found to be inhibited by the propeptide released from the unprocessed propernisine during degradation of the catalytic domain (Fig. 6F and Fig. 9). This presumably prevents further degradation of the less stable unprocessed propernisine molecules by this mature pernisine in the early stages of the maturation process when the unprocessed molecules predominate. Such inhibition of the early matured pernisine would therefore ensure that autoprocessing of the remaining unprocessed propernisine molecules proceeds completely. This would ensure that the final yield of mature pernisine molecules is not reduced by uncontrolled degradation of the unprocessed propernisine by the pre-existing mature protease.

One of the limitations of the present study is the use of propernisine with the active site mutations to simulate the unprocessed and autoprocessed propernisine forms. Since it is difficult to isolate a wild-type prosubtilase at the different processing stages, previous studies have also used active site mutants to obtain crystal structures of the unprocessed and autoprocessed prosubtilases (45, 58). However, it should be noted that these mutations themselves may partially affect protein structure and stability. Future studies would need to experimentally determine the structures of pernisine in complex with its propeptide to confirm the observations derived from the modeled structures and to identify the interactions responsible for the strong association between the two domains of propernisine.

### Conclusion

The results of this study demonstrate the selectivity in the interplay between the propeptide of pernisine and its cognate protease domain. Although pernisine is a highly active protease with prion-degrading activity, its propeptide domain is a difficult substrate for the pernisine catalytic domain and has been evolved to tightly regulate the maturation of propernisine in a high-temperature environment. The inhibitory activity of the propeptide presumably ensures that the prosubtilase is not activated before it is secreted into the extracellular space. Presumably, the exceptionally strong inhibition of subtilase by its propeptide, as observed with propernisine, is particularly important in hyperthermophilic organisms because high temperatures accelerate catalyzed processes such as maturation of prosubtilases.

## MATERIALS AND METHODS

### Site-directed mutagenesis, overexpression, and purification of recombinant proteins

To replace the catalytic Ser^261^ of pernisine with Cys, the codon encoding the corresponding Ser (TCG) in pernisine gene was replaced with TGC by the asymmetric overlap extension PCR method (59). For this, the PMCSG7-based expression plasmid encoding the codon-optimized propernisine gene from previous study (60) was used as template. Primer pairs P1-P2 and P3-P4 (Table 1) were used to amplify the upstream and downstream regions of the target TCG codon (including the TCG codon), respectively, in molar ratio 10 (P1, P4):1 (P2, P3). All DNA amplifications were carried out by Phusion DNA polymerase (Thermo Scientific). The amplified single strands were combined and hybridized at 65°C for 1 min. The complementary strands were elongated by further 5 min incubation at 72°C. The obtained double-stranded DNA fragment encoding the catalytic Ser→Cys propernisine mutant was cloned into the pMD204 vector (61) between the XhoI and EcoRI sites, as described in our previous study (12).

**TABLE 1:** Oligonucleotide sequences used in this study^*a*^

DNA primer	Sequence (from 5′ to 3′)	Purpose
P1	GAGCTGAATTCCGGTACGAAAATCGCCGCTATCGC	Forward primer for amplification of propernisine gene
P2	GCGGCGTAGCCAT**GCA**GGTGCCTGACAG	Introduction of S261C mutation in pernisine gene
P3	CTGTCAGGCACC**TGC**ATGGCTACGCCGC	Introduction of S261C mutation in pernisine gene
P4	GAGCTCTCGAGCCACTGGAGACAGCCGTTTGGACAG	Reverse primer for amplification of propernisine gene

^*a*^ The restriction sites are underlined. The codon encoding Cys is in bold text.

The pMD204-based expression plasmids encoding the His-tagged propernisine and its variants with the catalytic Ser replaced with Ala [prepared in reference (15)] or Cys (prepared in this study) were transformed into the competent *E. coli* BL21(DE3). The proteins were produced and isolated from *E. coli* periplasm as described in reference (12). The pernisine propeptide (PRO^P^) was produced and purified from the *E. coli* cytoplasm as described in reference (15). All proteins produced were dialyzed against 10 mM Tris (pH 8.0) overnight and stored at −80°C. The protein concentrations were determined by absorbance at 280 nm, using the extinction coefficients of 59,360 M^−1^ cm^−1^ and 1,490 M^−1^ cm^−1^ for the propernisine variants and PRO^P^, respectively. The yields of isolated propernisine and both propernisine mutants were ~5 mg per liter culture, whereas the yield of isolated PRO^P^ was ~20 mg/L culture.

### Preparation of unprocessed/autoprocessed propernisine and active pernisine

The isolated propernisine with the catalytic Ser^261^ replaced with Ala was regarded as the unprocessed propernisine (proPer^UP^). To prepare the autoprocessed propernisine form, the propernisine with the catalytic Ser^261^ replaced with Cys (propernisine^S261C^) was incubated in 10 mM Tris (pH 8.0), 10 mM CaCl_2_ at 90°C for 30 min. After centrifugation (18,000 × *g*, 10 min), supernatant was collected and further incubated for 120 min in dry bath system at 100°C. The active (mature) pernisine was prepared by incubation of nonmutated propernisine at 90°C for 5 h in 10 mM Tris (pH 8.0), 10 mM CaCl_2_. After the incubations, the autoprocessed propernisine (proPer^AP^) and the active pernisine were centrifuged at 18,000 × *g* for 15 min, and supernatants were stored at −80°C. The concentrations of proPer^UP^, proPer^AP^, and pernisine were calculated using absorbance at 280 nm and extinction coefficients of 59,360 M^−1^ cm^−1^ for proPer^UP^ and proPer^AP^, and 52,370 M^−1^ cm^−1^ for pernisine. For SEC and ITC experiments, pernisine was dialyzed against distilled water overnight, freeze dried, and stored at −80°C.

### Inhibitory activity of PRO^P^/proPer^UP^/proPer^AP^

The inhibition of pernisine, SubC, and PK by PRO^P^, proPer^UP^, and proPer^AP^ was determined using Suc-AAPF-*p*NA (N-succinyl-Ala-Ala-Pro-Phe-*p*-nitroanilide) as protease substrate. SubC, PK, and Suc-AAPF-*p*NA were purchased from Sigma-Aldrich. Separate 300-µL solutions of proteases (4 nM pernisine/4 nM PK/2 nM SubC) and 300-µL mixtures of substrate and inhibitor (0.6 mM Suc-AAPF-*p*NA and different concentrations of PRO^P^/proPer^UP^/proPer^AP^) were preincubated for 5 min at the same temperature as the following enzymatic reactions (25°C or 50°C). All protease solutions and substrate-inhibitor mixtures contained 20 mM Tris (pH 8.0), 1 mM CaCl_2_. The reactions were started by combining the protease solutions and substrate-inhibitor mixtures into quartz cuvettes (optical length 10 mm) that were preheated at the reaction temperature (25°C or 50°C). The final reaction mixtures (600 µL) contained 2 nM (pernisine and PK) or 1 nM (SubC) protease, 0.3 mM Suc-AAPF-*p*NA and different PRO^P^/proPer^UP^/proPer^AP^ concentrations (0–1 µM). The reactions were incubated in spectrophotometer (Cary 100 Bio UV-visible; Varian) at defined temperature (25°C or 50°C) and the *p*-nitroaniline released was measured over time by its absorbance at 410 nm.

### Determination of pernisine inhibition constants

Protease substrate Suc-AAPL-*p*NA (N-succinyl-Ala-Ala-Pro-Leu-*p*-nitroanilide; Sigma-Aldrich) was used to determine the apparent inhibition constant (*K*_i_′) of PRO^P^:pernisine complex. The enzymatic reactions were conducted as described above at 25°C or 50°C. The reaction mixtures (600 µL) contained 2 nM pernisine, 0.5 mM Suc-AAPL-*p*NA and different concentrations of PRO^P^ (0–8 nM) in 20 mM Tris (pH 8.0), 1 mM CaCl_2_. The progress curves were fitted to Equation 1 which describes the tight, slow-binding enzyme inhibition (62).


(1) $$A_{t}=v_{s}\cdot t+\frac{\left(1-\gamma \right)\cdot \left(v_{0}-v_{s}\right)}{k\cdot \gamma }\cdot ln\left(\frac{1-\gamma \cdot e^{-k\cdot t}}{1-\gamma }\right)+A_{0}$$

*A*_t_ and *A*_0_ stand for the absorbance of *p*-nitroaniline at time *t* and time 0, respectively, *v*_0_ and *v*_s_ are initial and steady-state velocities, respectively, and *k* is the progress curve rate constant. The value γ is defined in Equation 2.


(2) $$\gamma =\frac{E_{t}\cdot {\left(1-\frac{v_{s}}{v_{0}}\right)}^{2}}{I_{t}}$$

*E*_t_ and *I*_t_ correspond to total molar concentrations of pernisine (*E*_t_) and PRO^P^ (*I*_t_) in the reaction mixture. By fitting the progress curve data to Equation 1, the *v*_0_, *v*_s_, and *k* values at different PRO^P^ concentrations were obtained. The *K*_i_′ of PRO^P^:pernisine complex was determined from the slope of linear function described by Equation 3 (63).


(3) $$\frac{I_{t}}{\left(1-\frac{v_{s}}{v_{0}}\right)}=K_{i}^{\prime }\cdot \frac{v_{0}}{v_{s}}$$

### Size exclusion chromatography

Freeze-dried pernisine, SubC, and PK were dissolved in SEC buffer [20 mM Tris (pH 8.0), 150 mM NaCl, and 5 mM CaCl_2_] supplemented with 5 mM PMSF. After 60 min incubation at 4°C, samples were centrifuged at 18,000 × *g* for 10 min, and supernatants were collected. To validate inhibition of subtilases by PMSF, the supernatants were diluted 15,000-fold into 20 mM Tris (pH 8.0), 150 mM NaCl, 1 mM CaCl_2_, and 0.3 mM Suc-AAPF-*p*NA (final volume 600 µL) to the final subtilase concentration 2 nM. The release of *p*NA was measured by absorbance at 410 nm (*A*_410_) over time at 25°C. To prepare the samples for SEC, the PMSF-inhibited proteases were mixed either with PRO^P^ diluted in SEC buffer or with the same volume of SEC buffer and incubated at room temperature for 30 min. Final concentrations of PMSF-inhibited proteases were 0.15 mg/mL (4.3 µM pernisine, 5.5 µM SubC, and 5.2 µM PK) and the molar protease:PRO^P^ ratio was 1:1.25. After this preincubation, 400 µL samples were loaded onto HiLoad 16/600 Superdex 75 column (GE Healthcare) that was preequilibrated in SEC buffer. SEC was carried out using an NGC chromatography system (Bio-Rad) at 1 mL/min flow rate and the absorbance at 280 nm was continuously measured at the column outlet. To validate the autoprocessed state of proPer^AP^, proPer^AP^ was diluted from 1 mg/mL stock solution into 20 mM Tris (pH 8.0), 150 mM NaCl, and 5 mM CaCl_2_, to the final concentration 0.15 mg/mL and 400 µL were loaded onto HiLoad 16/600 Superdex 75 column. SEC was carried out as described above. The apparent molecular weights of protein species were estimated from chromatograms using the following protein standards: bovine serum albumin, albumin from chicken egg white (ovalbumin), trypsin and ribonuclease A (both from bovine pancreas). All protein standards were purchased from Sigma-Aldrich, dissolved in SEC buffer to 0.5–1 mg/mL and centrifuged at 18,000 × *g* for 10 min. About 400 µL supernatants were loaded onto the HiLoad 16/600 Superdex 75 column, and SEC was carried out as described above.

### Isothermal titration calorimetry

The ITC was conducted using the Nano ITC calorimeter (TA Instruments). Freeze-dried pernisine, SubC, and PK were dissolved in 20 mM Tris (pH 8.0), 10 mM CaCl_2_ with or without 5 mM PMSF and incubated for 60 min at 4°C. Afterward, proteins were centrifuged at 18,000 × *g,* and supernatants were dialyzed against 20 mM Tris (pH 8.0), 10 mM CaCl_2_ overnight. PRO^P^ was dialyzed against the same buffer. Dialyzed proteins were centrifuged at 18,000 × *g* and degassed under vacuum before the ITC analyses. The sample cell was filled with 5 µM solution of non-modified or PMSF-inhibited protease (pernisine/SubC/PK). Titration was performed by 19 individual 5 µL injections of 100 µM PRO^P^ every 900 s (titrations at 25°C) or 300 s (titrations at 50°C) into the protease solution. For blank experiments, 100 µM PRO^P^ was injected into the sample cell filled with dialysis buffer (20 mM Tris (pH 8.0), 10 mM CaCl_2_) without any protein. Baseline correction, peak integration, blank data subtraction, and fitting to the independent binding model were conducted using the NanoAnalyze software.

### PRO^P^ degradation assays

Proteases (pernisine/SubC/PK/trypsin) were diluted in 10 mM Tris (pH 8.0), 5 mM CaCl_2_. For degradation of the free pernisine propeptide, PRO^P^ was added into the protease solutions so that final concentrations of protease and the PRO^P^ were 2.5 and 25 µM, respectively. The 50 µL reactions were incubated in water baths at defined temperatures and stopped with TCA to the final concentration 10% (wt/vol). Stopped reactions were frozen at −20°C overnight. After thawing, the precipitated proteins were pelleted by 10 min centrifugation at 18,000 × *g* and 4°C, washed with 100% acetone, and centrifuged again as before. Pellets were dissolved in 4× sample buffer for SDS-PAGE, supplemented with 4 mM PMSF and 4 mM EDTA, boiled at 95°C for 5 min, and resolved using Tricine-SDS-PAGE with 15% polyacrylamide gels. The same protocol was followed for degradation of PRO^P^ complexed with pernisine catalytic domain (proPer^UP^ and proPer^AP^), except that the concentrations of proteases and proPer^UP^/proPer^AP^ were 0.8 µM and the volume of reaction mixtures was 200 µL.

### N-terminal sequencing

Proteins were resolved by Tricine-SDS-PAGE and electrotransferred from the 15% polyacrylamide gels onto polyvinylidene difluoride (PVDF) membranes (Thermo Scientific). The PVDF membranes were stained with Coomassie R-250, and the bands of interest were cut out. The N-termini were sequenced using the Edman degradation method (PPSQ-53A Gradient system; Shimadzu). The phenylthiohydantoin-amino acid derivatives were identified using a Wakosil PTH-GR (S-PSQ) column (Fujifilm Wako Pure Chemical Corporation).

### Protein structure modeling

Structures of protein complexes were modeled using ColabFold platform (v1.5.0) available at https://colab.research.google.com/github/sokrypton/ColabFold/blob/main/AlphaFold2.ipynb (28). ColabFold implements multiple sequence alignments generated by MMseqs2 and Alphafold2-multimer algorithm (64) to predict structures of protein complexes. Template search against the PDB70 database was enabled and the predicted models were relaxed using amber force fields. The input sequences were propeptides of pernisine (Ala^1^′-Met^68^′), SubC (Ala^1^′-Leu^76^′), PK (Ala^1^′-Asn^90^′), and the corresponding catalytic domains (Ser^12^-Ser^336^ for pernisine, Ala^1^-Gln^274^ for SubC, and Ala^1^-Ala^279^ for PK). The sequences were obtained at UniProt, with accession numbers Q9YFI3 (propernisine), P00780 (proSubC), and P06873 (proPK). Five models were generated for each input, with average per-residue confidence scores pLDDT between 94 and 98. The models were ranked by weighted combination of predicted TM-score and interface predicted TM-score (64) and the top-ranking model was used for analysis. The models were visualized by VMD software (29).

### Circular dichroism spectroscopy

The CD spectra of proPer^UP^ and proPer^AP^ were recorded with a CD spectrophotometer J-1500 (Jasco). The bandwidth was set at 1.0 nm, the scanning speed at 20 nm/min and temperature at 20°C. The CD spectra were scanned from 250 to 195 nm (far-UV range) and 320 to 250 nm (near-UV range). Quartz cuvettes with optical paths of 1 mm and 10 mm were used for measurements in the far-UV and near-UV ranges, respectively. Protein concentrations were 0.1 mg/mL (measurements in the far-UV range) and 0.35–0.5 mg/mL (measurements in near-UV range). All proteins were dissolved in 10 mM Tris (pH 8.0), 10 mM CaCl_2_. Mean residue weights of 102.4 were used to calculate the molar ellipticities of proPer^UP^ and proPer^AP^.

### Tryptophan spectrofluorimetry

The intrinsic tryptophan fluorescence spectra of proPer^UP^ and proPer^AP^ were collected with a fluorescence spectrophotometer Cary Eclipse (Varian). The excitation and emission slits were set at 5 nm. The scanning rate was 120 nm/min, with a signal averaging time of 0.5 s. The excitation wavelength was 293 nm and the emission spectra of proteins were scanned from 300 nm to 400 nm at 20°C. Stock protein solutions (0.5 mg/mL) in 10 mM Tris (pH 8.0), 10 mM CaCl_2_ were diluted 10-fold in buffers with different pH values. These buffers were 50 mM Na_2_HPO_4_-NaOH (pH 12.0), 50 mM glycine-NaOH (pH 10.0), 50 mM Tris-HCl (pH 8.0), 50 mM citrate (pH 4.0), and 50 mM glycine-HCl (pH 3.0 and pH 2.0). Final concentration of all proteins was 50 µg/mL.

### Differential scanning fluorimetry

The real-time PCR system QuantStudio 5 (Applied Biosystems) was used for temperature control and fluorescence measurements. The stock solutions of proPer^UP^ and proPer^AP^ (0.33 mg/mL) in 10 mM Tris (pH 8.0), 10 mM CaCl_2_ were diluted fivefold in 50 mM citrate buffer (pH 5.0). The same buffer was used to make the 20× solution of SYPRO Orange dye (Invitrogen) from the 5,000× concentrate. For DSF measurements, 15 µL diluted proteins were combined with 5 µL 20× SYPRO Orange. Final samples contained 1 µg protein and were prepared in triplicate. Samples were heated from 25°C to 99°C at the rate of 0.05°C per second. The SYPRO Orange fluorescence was measured over temperature increase, with excitation and emission wavelengths set at 470 and 587 nm, respectively. Raw data were processed and the melting temperatures were calculated using Protein Thermal Shift software (Applied Biosystems).

### Propernisine transactivation assays

The transactivation assays were conducted in 50 mM HEPES (pH 8.0), 10 mM CaCl_2_. Propernisine was mixed with active protease (SubC/PK/pernisine/trypsin) or equal volume of buffer, to the final volume of 400 µL. Final concentrations of propernisine and active proteases were 1 µM. Control reaction mixtures contained only the active protease (1 µM). The reaction mixtures were incubated at 40°C or 90°C in water bath. At the appropriate time intervals, 50 µL aliquots were withdrawn from each reaction mixture and frozen at −20°C to stop the reactions. Aliquots taken from the reaction mixtures that contained the mesophilic active protease (SubC/PK/trypsin) were exposed to 90°C for 1 min prior to freezing, to denature the active protease. All reactions were conducted in triplicate. The proteolytic activities in aliquots were determined in 96-well microtitre plates, using azocasein as substrate. For this, 5 µL sample was mixed with 95 µL azocasein (Sigma-Aldrich) in 50 mM HEPES (pH 8.0) for a final azocasein concentration of 1.5% (wt/vol). The reaction mixtures were incubated in the oven at 90°C for 20 min. Proteolysis was terminated by the addition of 30 µL 15% TCA and nondegraded azocasein was pelleted by centrifugation at 3,000 × *g* for 10 min. Afterward, 80 µL of the supernatants was collected and mixed with 30 µL 5 M NaOH. The absorbance of the released azo-dye was then determined at 440 nm. The absorbance values of control samples (active protease without the propernisine) were subtracted from the absorbance values of samples that initially contained propernisine with the active protease.

## Data Availability

All data described in the text are contained within the manuscript. No new nucleotide and amino acid sequences, strains, protein structures, etc., have been reported in this study. UniProt and PDB accession numbers for all mentioned proteins and structures have been given in the text.

## References

[1] Siezen RJ , Leunissen JAM . 1997. Subtilases: the superfamily of subtilisin-like serine proteases. Protein Sci 6:501–523. doi:10.1002/pro.5560060301 9070434 PMC2143677

[2] Yin S , Li M , Rao X , Yao X , Zhong Q , Wang M , Wang J , Peng Y , Tang J , Hu F , Zhao Y . 2016. Subtilisin-like protease-1 secreted through type IV secretion system contributes to high virulence of Streptococcus suis 2. Sci Rep 6. doi:10.1038/srep27369 PMC4897608 27270879

[3] Razzaq A , Shamsi S , Ali A , Ali Q , Sajjad M , Malik A , Ashraf M . 2019. Microbial proteases applications. Front Bioeng Biotechnol 7. doi:10.3389/fbioe.2019.00110 PMC6584820 31263696

[4] Shinde U , Thomas G . 2011. Insights from bacterial subtilases into the mechanisms of Intramolecular chaperone-mediated activation of furin. Methods Mol Biol 768:59–106. doi:10.1007/978-1-61779-204-5_4 21805238 PMC4300103

[5] Azrin NAM , Ali MSM , Rahman R , Oslan SN , Noor NDM . 2022. Versatility of subtilisin: a review on structure, characteristics, and applications. Biotechnol Appl Biochem 69:2599–2616. doi:10.1002/bab.2309 35019178

[6] Chen YJ , Inouye M . 2008. The intramolecular chaperone-mediated protein folding. Curr Opin Struct Biol 18:765–770. doi:10.1016/j.sbi.2008.10.005 18973809

[7] Shinde U , Inouye M . 1995. Folding mediated by an intramolecular chaperone: autoprocessing pathway of the precursor resolved via a substrate assisted catalysis mechanism. J Mol Biol 247:390–395. doi:10.1006/jmbi.1994.0147 7714895

[8] Ikemura H , Inouye M . 1988. In vitro processing of prosubtilisin produced in Escherichia coli *.* J Mol Biol 263:12959–12963. doi:10.1016/S0021-9258(18)37656-7 3047114

[9] Yabuta Y , Takagi H , Inouye M , Shinde U . 2001. Folding pathway mediated by an intramolecular chaperone: propeptide release modulates activation precision of prosubtilisin. J Biol Chem 276:44427–44434. doi:10.1074/jbc.M107573200 11577106

[10] Uehara R , Takano K , Kanaya S , Koga Y , Brahmachari G . 2017. Hyperthermophilic Subtilisin-like proteases from *Thermococcus Kodakarensis* , p 81–117. In Biotechnology of microbial enzymes: Production, Biocatalysis and industrial applications. Academic Press, Cambridge, MA.

[11] Gao X , Zeng J , Yi H , Zhang F , Tang B , Tang XF . 2017. Four inserts within the catalytic domain confer extra stability and activity to hyperthermostable pyrolysin from Pyrococcus furiosus. Appl Environ Microbiol 83:e03228-16. doi:10.1128/AEM.03228-16 28003199 PMC5311392

[12] Bahun M , Hartman K , Poklar Ulrih N . 2020. Periplasmic production of pernisine in Escherichia coli and determinants for its high thermostability. Appl Microbiol Biotechnol 104:7867–7878. doi:10.1007/s00253-020-10791-w 32734388

[13] Snajder M , Vilfan T , Cernilec M , Rupreht R , Popović M , Juntes P , Serbec VČ , Ulrih NP . 2012. Enzymatic degradation of Prp^sc^ by a protease secreted from Aeropyrum pernix K1. PLoS One 7:e39548. doi:10.1371/journal.pone.0039548 22761822 PMC3386259

[14] Šnajder M , Carrillo Rincón AF , Magdevska V , Bahun M , Kranjc L , Paš M , Juntes P , Petković H , Poklar Ulrih N . 2019. Extracellular production of the engineered thermostable protease pernisine from Aeropyrum pernix K1 in Streptomyces rimosus. Microb Cell Fact 18:196. doi:10.1186/s12934-019-1245-3 31699090 PMC6839199

[15] Bahun M , Šnajder M , Turk D , Poklar Ulrih N . 2020. Insights into the maturation of pernisine, a subtilisin-like protease from the hyperthermophilic archaeon Aeropyrum pernix. Appl Environ Microbiol 86:17. doi:10.1128/AEM.00971-20 PMC7440795 32561587

[16] Ikemura H , Takagi H , Inouye M . 1987. Requirement of prosequence for the production of active subtilisin E. J Biol Chem 262:7859–7864. 3108260

[17] Shinde U , Inouye M . 1996. Propeptide-mediated folding in subtilisin: the intramolecular chaperone concept. Adv Exp Med Biol 379:147–154. doi:10.1007/978-1-4613-0319-0_16 8796319

[18] Tanaka S , Takeuchi Y , Matsumura H , Koga Y , Takano K , Kanaya S . 2008. Crystal structure of Tk-subtilisin folded without propeptide: requirement of propeptide for acceleration of folding. FEBS Lett 582:3875–3878. doi:10.1016/j.febslet.2008.10.025 18951896

[19] Pulido MA , Koga Y , Takano K , Kanaya S . 2007. Directed evolution of Tk-subtilisin from a hyperthermophilic archaeon: identification of a single amino-acid substitution responsible for low-temperature adaptation. Prot Eng Des Sel 20:143–153. doi:10.1093/protein/gzm006 17351019

[20] Huang HW , Chen WC , Wu CY , Yu HC , Lin WY , Chen ST , Wang KT . 1997. Kinetic studies of the inhibitory effects of propeptides subtilisin BPN' and carlsberg to bacterial serine proteases. Prot Eng Des Sel 10:1227–1233. doi:10.1093/protein/10.10.1227 9488148

[21] Eder J , Rheinnecker M , Fersht AR . 1993. Folding of subtilisin BPN’: role of the prosequence. J Mol Biol 233:293–304. doi:10.1006/jmbi.1993.1507 8377204

[22] Pulido M , Saito K , Tanaka S-I , Koga Y , Morikawa M , Takano K , Kanaya S . 2006. Ca2+ dependent maturation of subtilisin from a hyperthermophilic archaeon, Thermococcus kodakaraensis: the propeptide is a potent inhibitor of the mature domain but is not required for its folding. Appl Environ Microbiol 72:4154–4162. doi:10.1128/AEM.02696-05 16751527 PMC1489632

[23] Gallagher T , Gilliland G , Wang L , Bryan P . 1995. The prosegment-subtilisin BPN′ complex: crystal structure of a specific “foldase". Structure 3:907–914. doi:10.1016/S0969-2126(01)00225-8 8535784

[24] Tanaka S , Saito K , Chon H , Matsumura H , Koga Y , Takano K , Kanaya S . 2007. Crystal structure of unautoprocessed precursor of subtilisin from a hyperthermophilic archaeon: evidence for Ca2+^-I^Nduced folding. J Biol Chem 282:8246–8255. doi:10.1074/jbc.M610137200 17237225

[25] Sievers F , Wilm A , Dineen D , Gibson TJ , Karplus K , Li W , Lopez R , McWilliam H , Remmert M , Söding J , Thompson JD , Higgins DG . 2011. Fast, scalable generation of high‐quality protein multiple sequence alignments using Clustal Omega. Mol Syst Biol 7. doi:10.1038/msb.2011.75 PMC3261699 21988835

[26] Goujon M , McWilliam H , Li W , Valentin F , Squizzato S , Paern J , Lopez R . 2010. A new bioinformatics analysis tools framework at EMBL-EBI. Nucleic Acids Res 38:W695–9. doi:10.1093/nar/gkq313 20439314 PMC2896090

[27] Waterhouse AM , Procter JB , Martin DMA , Clamp M , Barton GJ . 2009. Jalview version 2—a multiple sequence alignment editor and analysis workbench. Bioinformatics 25:1189–1191. doi:10.1093/bioinformatics/btp033 19151095 PMC2672624

[28] Mirdita M , Schütze K , Moriwaki Y , Heo L , Ovchinnikov S , Steinegger M . 2022. Colabfold: making protein folding accessible to all. Nat Methods 19:679–682. doi:10.1038/s41592-022-01488-1 35637307 PMC9184281

[29] Humphrey W , Dalke A , Schulten K . 1996. VMD: Visual molecular dynamics. J Mol Graph 14:33–38, doi:10.1016/0263-7855(96)00018-5 8744570

[30] Kelly SM , Price NC . 2000. The use of circular dichroism in the investigation of protein structure and function. Curr Protein Pept Sci 1:349–384. doi:10.2174/1389203003381315 12369905

[31] Li Y , Hu Z , Jordan F , Inouye M . 1995. Functional analysis of the propeptide of subtilisin E as an intramolecular chaperone for protein folding: refolding and inhibitory abilities of propeptide mutants. J Biol Chem 270:25127–25132. doi:10.1074/jbc.270.42.25127 7559646

[32] Fox T , de Miguel E , Mort JS , Storer AC . 1992. Potent slow-binding inhibition of cathepsin B by its propeptide. Biochemistry 31:12571–12576. doi:10.1021/bi00165a005 1472493

[33] Guay J , Falgueyret JP , Ducret A , Percival MD , Mancini JA . 2000. Potency and selectivity of inhibition of cathepsin K, L and S by their respective propeptides. Eur J Biochem 267:6311–6318. doi:10.1046/j.1432-1327.2000.01730.x 11012686

[34] Subbian E , Yabuta Y , Shinde UP . 2005. Folding pathway mediated by an intramolecular chaperone: intrinsically unstructured propeptide modulates stochastic activation of subtilisin. J Mol Biol 347:367–383. doi:10.1016/j.jmb.2005.01.028 15740747

[35] Tanaka S , Matsumura H , Koga Y , Takano K , Kanaya S . 2009. Identification of the interactions critical for propeptide-catalyzed folding of Tk-subtilisin. J Mol Biol 394:306–319. doi:10.1016/j.jmb.2009.09.028 19766655

[36] Zhu H , Xu BL , Liang X , Yang YR , Tang XF , Tang B . 2013. Molecular basis for auto- and hetero-catalytic maturation of a thermostable subtilase from thermophilic Bacillus sp. J Biol Chem 288:34826–34838. doi:10.1074/jbc.M113.498774 24145031 PMC3843095

[37] Okubo A , Li M , Ashida M , Oyama H , Gustchina A , Oda K , Dunn BM , Wlodawer A , Nakayama T . 2006. Processing, catalytic activity and crystal structures of kumamolisin-as with an engineered active site. FEBS J 273:2563–2576. doi:10.1111/j.1742-4658.2006.05266.x 16704427

[38] Radisky E.S , Koshland DE . 2002. A clogged gutter mechanism for protease inhibitors. Proc Natl Acad Sci U S A 99:10316–10321. doi:10.1073/pnas.112332899 12142461 PMC124911

[39] Radisky Evette S , Kwan G , Karen Lu C-J , Koshland DE Jr . 2004. Binding, proteolytic, and crystallographic analyses of mutations at the protease−inhibitor interface of the subtilisin BPN‘/chymotrypsin inhibitor 2 complex. Biochemistry 43:13648–13656. doi:10.1021/bi048797k 15504027

[40] Meyer M , Leptihn S , Welz M , Schaller A . 2016. Functional characterization of propeptides in plant subtilases as intramolecular chaperones and inhibitors of the mature protease. J Biol Chem 291:19449–19461. doi:10.1074/jbc.M116.744151 27451395 PMC5016683

[41] Marie-Claire C , Yabuta Y , Suefuji K , Matsuzawa H , Shinde U . 2001. Folding pathway mediated by an intramolecular chaperone: the structural and functional characterization of the aqualysin I propeptide. J Mol Biol 305:151–165. doi:10.1006/jmbi.2000.4233 11114254

[42] Yabuta Y , Subbian E , Oiry C , Shinde U . 2003. Folding pathway mediated by an intramolecular chaperone: a functional peptide chaperone designed using sequence databases. J Biol Chem 278:15246–15251. doi:10.1074/jbc.M212003200 12582173

[43] Strausberg S , Alexander P , Wang L , Schwarz F , Bryan P . 1993. Catalysis of a protein folding reaction: thermodynamic and kinetic analysis of subtilisin BPN' interactions with its propeptide fragment. Biochemistry 32:8112–8119. doi:10.1021/bi00083a009 8347611

[44] Shinde U , Li Y , Chatterjee S , Inouye M . 1993. Folding pathway mediated by an intramolecular chaperone. Proc Natl Acad Sci U S A 90:6924–6928. doi:10.1073/pnas.90.15.6924 8346198 PMC47047

[45] Tanaka S-I , Matsumura H , Koga Y , Takano K , Kanaya S . 2007. Four new crystal structures of Tk-subtilisin in unautoprocessed, autoprocessed and mature forms: insight into structural changes during maturation. J Mol Biol 372:1055–1069. doi:10.1016/j.jmb.2007.07.027 17706669

[46] Kojima S , Minagawa T , Miura K . 1998. Tertiary structure formation in the propeptide of subtilisin BPN′ by successive amino acid replacements and its close relation to function. J Mol Biol 277:1007–1013. doi:10.1006/jmbi.1998.1671 9571018

[47] Pulido MA , Tanaka S , Sringiew C , You D-J , Matsumura H , Koga Y , Takano K , Kanaya S . 2007. Requirement of left-handed glycine residue for high stability of the Tk-subtilisin propeptide as revealed by mutational and crystallographic analyses. J Mol Biol 374:1359–1373. doi:10.1016/j.jmb.2007.10.030 17988685

[48] Yuzaki K , Sanda Y , You DJ , Uehara R , Koga Y , Kanaya S . 2013. Increase in activation rate of pro-Tk-subtilisin by a single nonpolar-to-polar amino acid substitution at the hydrophobic core of the propeptide domain. Protein Sci 22:1711–1721. doi:10.1002/pro.2371 24115021 PMC3843626

[49] Daugherty AB , Muthu P , Lutz S . 2012. Novel protease inhibitors via computational redesign of subtilisin BPN′ propeptide. Biochemistry 51:8247–8255. doi:10.1021/bi300832v 23009354

[50] Nakagawa M , Ueyama M , Tsuruta H , Uno T , Kanamaru K , Mikami B , Yamagata H . 2010. Functional analysis of the cucumisin propeptide as a potent inhibitor of its mature enzyme. J Biol Chem 285:29797–29807. doi:10.1074/jbc.M109.083162 20639575 PMC2943254

[51] Sotokawauchi A , Kato-Murayama M , Murayama K , Hosaka T , Maeda I , Onjo M , Ohsawa N , Kato D-I , Arima K , Shirouzu M . 2017. Structural basis of cucumisin protease activity regulation by its propeptide. J Biochem 161:45–53. doi:10.1093/jb/mvw053 27616715

[52] Jean L , Hackett F , Martin SR , Blackman MJ . 2003. Functional characterization of the propeptide of Plasmodium falciparum subtilisin-like protease-1. J Biol Chem 278:28572–28579. doi:10.1074/jbc.M303827200 12764150

[53] Uehara R , Ueda Y , You DJ , Koga Y , Kanaya S . 2013. Accelerated maturation of Tk-subtilisin by a Leu→Promutation at the C-terminus of the propeptide, which reduces the binding of the propeptide to Tk-subtilisin. FEBS J 280:994–1006. doi:10.1111/febs.12091 23237738

[54] Kojima S , Deguchi M , Miura K . 1999. Involvement of the C-terminal region of yeast proteinase B inhibitor 2 in its inhibitory action. J Mol Biol 286:775–785. doi:10.1006/jmbi.1998.2498 10024450

[55] Kojima S , Hisano Y , Miura K . 2001. Alteration of inhibitory properties of Pleurotus ostreatus proteinase A inhibitor 1 by mutation of its C-terminal region. Biochem Biophys Res Commun 281:1271–1276. doi:10.1006/bbrc.2001.4515 11243873

[56] Kojima S , Minagawa T , Miura K . 1997. The propeptide of subtilisin BPN′ as a temporary inhibitor and effect of an amino acid replacement on its inhibitory activity. FEBS Lett 411:128–132. doi:10.1016/s0014-5793(97)00678-9 9247157

[57] Uehara R , Takeuchi Y , Tanaka S , Takano K , Koga Y , Kanaya S . 2012. Requirement of Ca2+ ions for the hyperthermostability of Tk-subtilisin from Thermococcus Kodakarensis. Biochemistry 51:5369–5378. doi:10.1021/bi300427u 22686281

[58] Jain SC , Shinde U , Li Y , Inouye M , Berman HM . 1998. The crystal structure of an autoprocessed Ser221Cys-subtilisin E-propeptide complex at 2.0 Å resolution. J Mol Biol 284:137–144. doi:10.1006/jmbi.1998.2161 9811547

[59] Xiao YH , Pei Y . 2011. Asymmetric overlap extension PCR method for site-directed mutagenesis. Methods Mol Biol 687:277–282. doi:10.1007/978-1-60761-944-4_20 20967616

[60] Šnajder M , Mihelič M , Turk D , Ulrih NP . 2015. Codon optimisation is key for pernisine expression in Escherichia coli. PLoS One 10:e0123288. doi:10.1371/journal.pone.0123288 25856104 PMC4391949

[61] Skrlj N , Erculj N , Dolinar M . 2009. A versatile bacterial expression vector based on the synthetic biology plasmid pSB1. Protein Expr Purif 64:198–204. doi:10.1016/j.pep.2008.10.019 19027858

[62] Williams JW , Morrison JF , Duggleby RG . 1979. Methotrexate, a high-affinity pseudosubstrate of dihydrofolate reductase. Biochemistry 18:2567–2573. doi:10.1021/bi00579a021 36135

[63] Szedlacsek SE , Duggleby RG . 1995. Kinetics of slow and tight-binding inhibitors. Methods Enzymol 249:144–180. doi:10.1016/0076-6879(95)49034-5 7791610

[64] Evans R , O’Neill M , Pritzel A , Antropova N , Senior A , Green T , Žídek A , Bates R , Blackwell S , Yim J , Ronneberger O , Bodenstein S , Zielinski M , Bridgland A , Potapenko A , Cowie A , Tunyasuvunakool K , Jain R , Clancy E , Kohli P , Jumper J , Hassabis D . 2021. Protein complex prediction with AlphaFold-Multimer. bioRxiv. doi:10.1101/2021.10.04.463034

